# Blood lipid levels mediating the effects of sex hormone-binding globulin on coronary heart disease: Mendelian randomization and mediation analysis

**DOI:** 10.1038/s41598-024-62695-8

**Published:** 2024-05-25

**Authors:** Juntao Yang, Jiedong Zhou, Hanxuan Liu, Jinjin Hao, Songqing Hu, Peipei Zhang, Haowei Wu, Yefei Gao, Weiliang Tang

**Affiliations:** 1 School of Medicine, Shaoxing University, Shaoxing, Zhejiang China; 2 Department of Cardiology, Shaoxing People’s Hospital, 568 Zhongxing North Road, Shaoxing, 312000 Zhejiang China; 3 School of Medicine, Zhejiang University, Hangzhou, Zhejiang China; 4 The Second Clinical Medical College, Zhejiang Chinese Medical University, Hangzhou, Zhejiang China

**Keywords:** Sex hormone-binding globulin, Blood lipid levels, Coronary heart disease, Cardiovascular disease, Mendelian randomization, Biomarkers, Cardiology, Molecular medicine

## Abstract

Observational studies indicate that serum sex hormone-binding globulin (SHBG) levels are inversely correlated with blood lipid levels and coronary heart disease (CHD) risk. Given that dyslipidemia is an established risk factor for CHD, we aim to employ Mendelian randomization (MR) in conjunction with mediation analysis to confirm the mediating role of blood lipid levels in the association between SHBG and CHD. First, we assessed the causality between serum SHBG levels and five cardiovascular diseases using univariable MR. The results revealed causality between SHBG levels and reduced risk of CHD, myocardial infarction, as well as hypertension. Specifically, the most significant reduction was observed in CHD risk, with an odds ratio of 0.73 (95% CI 0.63–0.86) for each one-standard-deviation increase in SHBG. The summary-level data of serum SHBG levels and CHD are derived from a sex-specific genome-wide association study (GWAS) conducted by UK Biobank (sample size = 368,929) and a large-scale GWAS meta-analysis (60,801 cases and 123,504 controls), respectively. Subsequently, we further investigated the mediating role of blood lipid level in the association between SHBG and CHD. Mediation analysis clarified the mediation proportions for four mediators: high cholesterol (48%), very low-density lipoprotein cholesterol (25.1%), low-density lipoprotein cholesterol (18.5%), and triglycerides (44.3%). Summary-level data for each mediator were sourced from the UK Biobank and publicly available GWAS. The above results confirm negative causality between serum SHBG levels and the risk of CHD, myocardial infarction, and hypertension, with the causal effect on reducing CHD risk largely mediated by the improvement of blood lipid profiles.

## Introduction

Sex hormone-binding globulin (SHBG) is a circulating plasma protein synthesized by liver cells. Traditional perspectives suggest that its primary physiological function is to regulate the bioavailability and metabolic clearance of sex hormones by specifically binding to them. It can also exert direct biological effects on tissues and cells dependent on sex hormones^1^. However, in recent years, in-depth research on SHBG has generated new insights, suggesting that it is closely associated with metabolic-related phenotypes and diseases such as blood lipids, hepatic fat content, obesity, insulin resistance, and diabetes^2–5^. Previous research has indicated association between higher serum SHBG levels and favorable lipid profile^6–8^, but traditional observational studies are susceptible to various biases^9^. Therefore, these findings need to be further verified by evidence-based medicine methods with higher level of evidence. Additionally, other studies have shown inverse association between serum SHBG levels and the risk of coronary heart disease (CHD)^10^, but the mechanism is not clear. The lipid infiltration hypothesis is currently the most widely accepted theory explaining the mechanism of atherosclerosis^11^. Of course, not only this process involves lipid accumulation, but also molecular processes such as excessive inflammatory response play an important role^12^. Therefore, dyslipidemia has been considered to be one of the most important risk factors for CHD^13^. In light of this, it is reasonable to speculate that the improvement of lipid profiles may play a crucial mediating role in the association between SHBG and CHD, and this hypothesis can be validated through joint mediation analysis.

**Figure 1:**
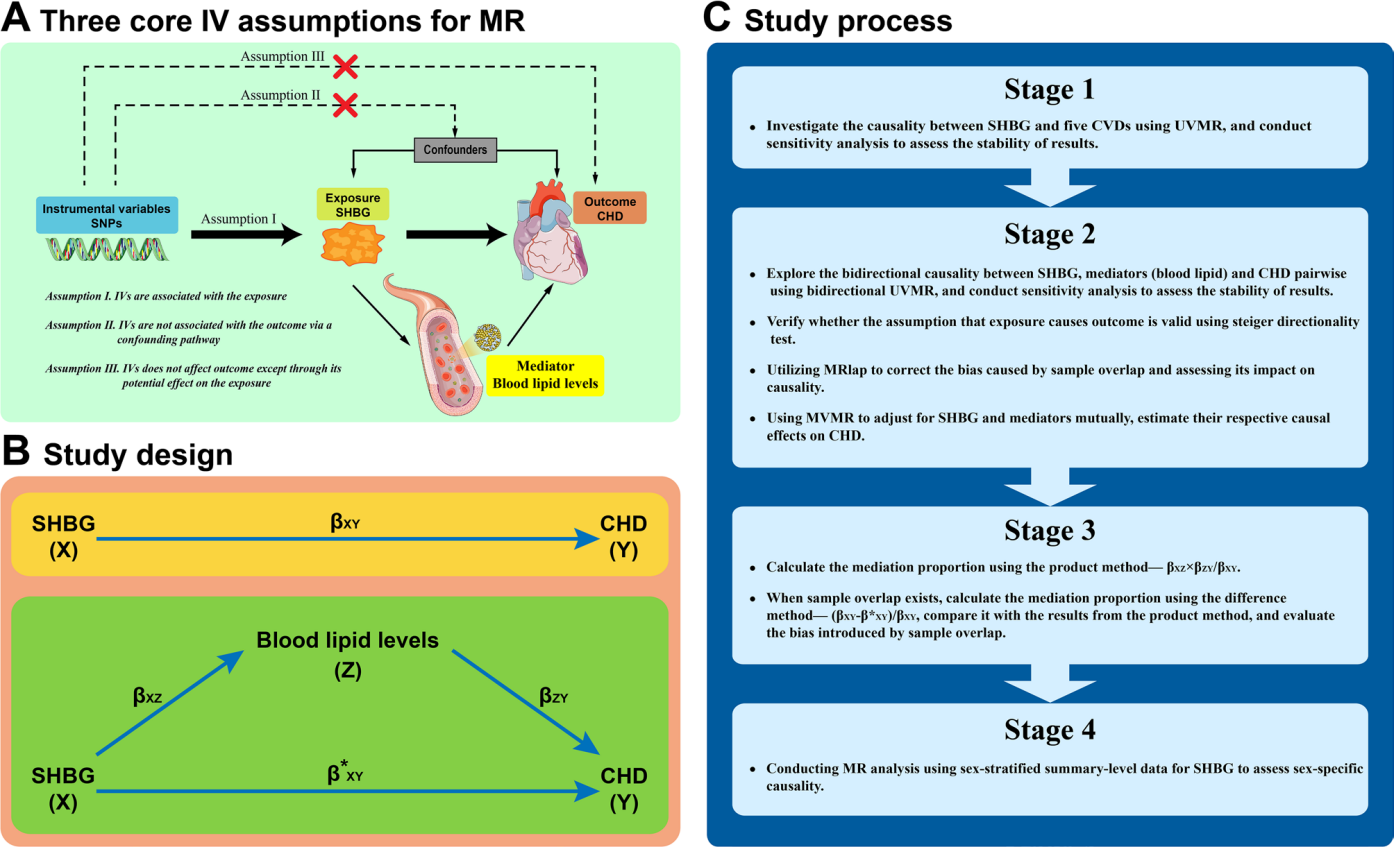
Overview of this study and illustrative diagram of the MR core IV assumption. (**A**) The illustrative diagram of three core instrumental variable assumptions in MR, with the incorporation of the mediators relevant to this study. (**B**) X, Y and Z represent exposure, mediator, and outcome, respectively. β_XY_ and β_XZ_ are derived using UVMR, while β^*^_XY_ and β_ZY_ are derived using MVMR with mutual adjustments for SHBG and mediator. (**C**) The study process is divided into four stages, corresponding to the "Study design and process" section. *SHBG* sex hormone-binding globulin, *CHD* coronary heart disease, *CVDs* cardiovascular diseases, *MR* Mendelian randomization, *UVMR* univariable Mendelian randomization, *MVMR* multivariable Mendelian randomization, *IV* instrumental variable, *SNPs* single-nucleotide polymorphisms.

Mendelian randomization (MR) is a novel causal inference method that has been increasingly employed in recent years. Its fundamental principle involves using genetic variation as instrumental variables (IVs) to predict the corresponding exposure. If populations with these genetic variations exhibit higher occurrence of the relevant outcome, it can establish a causality between exposure and outcome^14^. Since individual genetic variations are randomly allocated at conception and not influenced by postnatal factors, MR can overcome issues of confounders and reverse causality commonly encountered in traditional observational studies^15^. Additionally, when traditional epidemiological studies are costly and exposure are difficult to measure, MR can serve as a valuable technique^15^. Of course, this method has some limitations. The use of genetic variations as IVs in MR studies must satisfy three core instrumental variable (IV) assumptions: the relevance assumption, independence assumption, and exclusion restriction assumption (Fig. 1A)^16^. Because the IV assumptions are difficult to be fully verified, MR must be used with caution for causal inference. If these three assumptions are not entirely met, the analysis results may be subject to bias^16^.

Using MR, we aimed to further investigate the causality of serum SHBG levels on blood lipid levels and CHD risk. Mediation analysis was also introduced to demonstrate the mediating role of blood lipid levels in the causality between serum SHBG levels and CHD, and to determine the mediation proportion.

## Methods

### Study design and process

First, using univariable Mendelian randomization (UVMR) to assess the causality between serum SHBG levels and five cardiovascular diseases (CVDs), it was found that SHBG has the most significant causality with a reduced risk of CHD. Consequently, further research was conducted to investigate whether SHBG affects CHD through mediator (blood lipid levels). Second, we conducted bidirectional UVMR between serum SHBG levels and blood lipid levels, as well as between blood lipid levels and CHD. If there is a potential reverse causality, we employed the Steiger directionality test to assess the validity of the forward causality^17^. Subsequently, we performed multivariable Mendelian randomization (MVMR) with adjustment for SHBG to evaluate the direct effect of blood lipid levels on CHD. Third, we calculate the mediation proportion using the product method^18,19^. When there was sample overlap between the genome-wide association study (GWAS) on exposure and the GWAS on mediator, we then employ the difference method to compute the mediation proportion^18^. Fourth, conduct sex-specific analysis. The entire study design and process are illustrated in the Fig. 1B,C. This study strictly adheres to the reporting guidelines of Strengthening the Reporting of Observational Studies in Epidemiology Using Mendelian Randomization (STROBE-MR)^20,21^.

This study uses publicly available GWAS summary-level data, and thus, ethical approval is not required. Ethical approval and participant informed consent for the original GWAS can be found in the referenced GWAS publications and in the official websites of the respective databases.

### Data sources

The summary-level data stratified by gender for serum SHBG levels were derived from the largest sex-specific GWAS conducted by the UK Biobank, with BMI adjustments. This GWAS was also replicated in three independent studies (CHARGE Consortium, Twins UK and EPIC-Norfolk)^22^. The summary-level data of total cholesterol (TC) were obtained from 23 studies conducted by the Global Lipids Genetics Consortium, involving 94,595 participants of European ancestry^23^. The summary-level data of triglycerides (TG) and high density-lipoprotein cholesterol (HDL-C) were obtained from a GWAS conducted by the Within Family Consortium in 2022^24^. We acquired these data from the IEU OpenGWAS project (https://gwas.mrcieu.ac.uk/datasets), with the GWAS IDs being ieu-b-4850 and ieu-b-4844, respectively. Within Family GWAS can significantly mitigate the false genetic variation-phenotype association bias induced by transmission ratio distortion, population stratification, and assortative mating, thus providing more reliable causal effect estimates^25^. The summary-level data of low density-lipoprotein cholesterol (LDL-C) were derived from a GWAS involving 431,167 individuals from the UK Biobank^26^. The summary-level data of very low density-lipoprotein cholesterol (VLDL-C) were obtained from a GWAS conducted by Nightingale Health on 500,000 plasma samples from the UK Biobank^27^. The summary-level data for high cholesterol and hypercholesterolemia were obtained through the analysis of the UK Biobank genetic dataset using the GWAS pipeline developed by the Medical Research Council Integrative Epidemiology Unit at the University of Bristol^28^. This pipeline includes steps such as data quality control, genotype preprocessing, phenotype preprocessing, association analysis, and result integration^28^. The summary-level data of CHD were obtained from a large-scale GWAS meta-analysis conducted by the CARDIoGRAMplusC4D consortium, involving 48 studies and a total of 184,305 participants^29^. The summary-level data of myocardial infarction (MI) were derived from a subgroup analysis of this GWAS meta-analysis, accounting for approximately 70% of the total cases^29^. The summary-level data of hypertension, heart failure, and atrial fibrillation and flutter were all obtained from the FinnGen consortium R9 release^30^. The Finnish consortium defines these diseases using the codes from the International Classification of Diseases 8th, 9th, and 10th editions.

All details regarding the GWAS summary-level data and related databases mentioned above can be found in the Supplementary Tables S1–S4 and Supplementary Methods S1. The units for GWAS summary-level data across all phenotypes, the utilized regression models, and statistical transformations applied to certain data, are reported in the Supplementary Methods S2.

### Genetic instruments selection

The IVs [i.e., single nucleotide polymorphisms (SNPs)] associated with SHBG, blood lipid levels, and CVDs will be selected through the following steps. First, SNPs need to reach the genome-wide significance threshold (P < 5 × 10^–8^). Second, SNPs must be independent of each other, and we set the threshold for linkage disequilibrium as r^2^ < 0.001 and clumping window > 10,000 kb. Third, we also calculated the F-statistic for each SNP to assess their strength of association with the phenotype, and SNPs with an F-statistic less than 10 will be considered weak IVs and excluded^31^. The F-statistic is calculated as follows: $F=N\times \frac{{R}^{2}}{1-{R}^{2}}$, where R^2^ is the variability explained by each SNP, and N is the GWAS sample size^32^. To calculate R^2^ for the extended 10 SNPs, we used the following formula: ${R}^{2}=\frac{{\beta }^{2}}{{\beta }^{2}+N\times {SE}^{2}}$, where β is the estimated genetic effect and SE is the standard error of the genetic effect^33^. Before conducting MR analysis, all datasets need to be harmonized to align the direction of the allele of the SNPs associated with exposure and outcome and to exclude palindromic and inappropriate SNPs.

### UVMR and MVMR analysis

We conducted UVMR to assess the total causal effect of SHBG on five CVDs. Additionally, we performed bidirectional UVMR to evaluate the reciprocal causal effects among SHBG, blood lipid levels, and CHD. Furthermore, we conducted sex-specific analysis of the causality between SHBG and CHD using sex-stratified summary-level data. Inverse variance weighted (IVW) method is used as the primary UVMR analysis method, and the choice of the analytical model depends on the heterogeneity situation. This method is the meta-analysis of Wald ratios for each SNP^34^, thus providing the most accurate estimate of causal effects. However, it assumes that all IVs are valid, making it susceptible to potential horizontal pleiotropy^35,36^. Therefore, we employed the weighted median, MR-Egger, and MR-Pleiotropy Residual Sum and Outlier (MR-PRESSO) method as supplementary analytical methods. The weighted median method provides consistent estimates of causal effects when at least 50% of the weight comes from valid IVs^37^. The MR-Egger method can detect the presence of pleiotropy in MR analysis results and provide effect estimates that are not influenced by violations of IV assumptions^38,39^. The MR-PRESSO method can detect potentially pleiotropic SNPs (i.e., outliers) and assess whether removing outliers affects the effect estimates^40^. We further assessed the direct effects of SHBG and blood lipid levels on the CHD after mutual adjustment using MVMR^18^, with the IVW being the sole analytical method.

For continuous outcomes, the results will be presented using regression coefficient (i.e., β) and their 95% confidence interval (CI). For dichotomous outcomes, the main results will be reported in terms of odds ratio (OR) and its 95% CI.

### Mediation MR analysis

We employed a two-step MR to assess whether blood lipid levels act as mediators in mediating the risk reduction of CHD associated with SHBG. The two-step MR produced the following four effect estimates: (1) The total effect of SHBG on CHD (β_XY_); (2) The total effect of SHBG on the mediators (β_XZ_); (3) The direct effect of SHBG on CHD (β^*^_XY_); (4) The effect of mediators on CHD adjusting for SHBG (β_ZY_) (Fig. 1B). The indirect effect was calculated using the coefficient product method: Indirect effect = β_XZ_ × β_ZY_. And the mediation proportion was computed by dividing the indirect effect by β_XY_^18,19^. When there was sample overlap between the GWAS on exposure and the GWAS on mediators, the indirect effect can be calculated using the difference method: Indirect effect = β_XY_ − β^*^_XY_^18^. Because this method does not require the effect estimates of exposure on mediators, the resulting indirect effect is theoretically unaffected by the bias introduced by sample overlap. Next, we compare the mediation proportions calculated using the two methods. The 95% CI for mediation proportion are calculated using the delta method^41^ and bootstrap method.

### Sensitivity analyses

MR-Egger intercept test was used to detect potential horizontal pleiotropy in the results of UVMR^39^, while the Cochran's Q test was used to assess heterogeneity. When the p-values calculated by these two methods are less than 0.05, it indicates the presence of pleiotropy and heterogeneity, respectively. If horizontal pleiotropy is absent, it can provide evidence for the validity of assumption III. Considering the possibility of reverse causality and its impact on the reliability of the results, we used the Steiger directionality test to validate the validity of the forward causality^17^. Due to concerns that sample overlap between exposure and mediators may inflate causality and increase type I error rates^42^, we used MRlap to correct for bias caused by sample overlap^43^, and evaluated its impact on the results (Supplementary Methods S3). We used MR visualization methods to visually present the results of the UVMR. The scatter plot depicted the effect estimates and intercept of the MR-Egger method, while the funnel plot displayed heterogeneity. Additionally, leave-one-out analysis was conducted to assess the robustness of the results. In the MVMR analysis, we employed the "pleiotropy_mvmr" and "strength_mvmr" functions from the "MVMR" package to assess heterogeneity and the overall F-statistic for individual variable's SNPs, respectively.

Based on the aforementioned methods, the MR analysis results are defined as having a significant causality only if the following conditions are met: (1) The p-value of the IVW method is less than 0.05; (2) The effect estimates from all four MR analysis methods show consistent directions; (3) The MR-Egger intercept test suggests the absence of horizontal pleiotropy; (4) The Steiger directionality test confirms the establishment of forward causality.

In our study, we utilized R packages “TwoSampleMR,” “MRPRESSO,” “MRlap” and “MVMR” in R software version 4.2.2 (https://www.r-project.org/) for all statistical analyses. All p values in this study are two-sided, and the statistical significance was set at < 0.05.

### Ethics declarations

The ethical approval and informed consent from participants for the original GWAS are available in the cited GWAS publications and on the official websites of the relevant databases.

## Results

### Genetic instruments

The detailed information for genetic IVs used in UVMR and MVMR, along with their F-statistics, is reported in Supplementary Table S5–S25. All F-statistics are greater than 10, indicating the absence of weak instrument bias. Since less than 20% of the IVs were missing in the outcome summary-level data (except for TC), we did not search for proxy SNPs.

### Effects of SHBG on multiple CVDs

**Figure 2:**
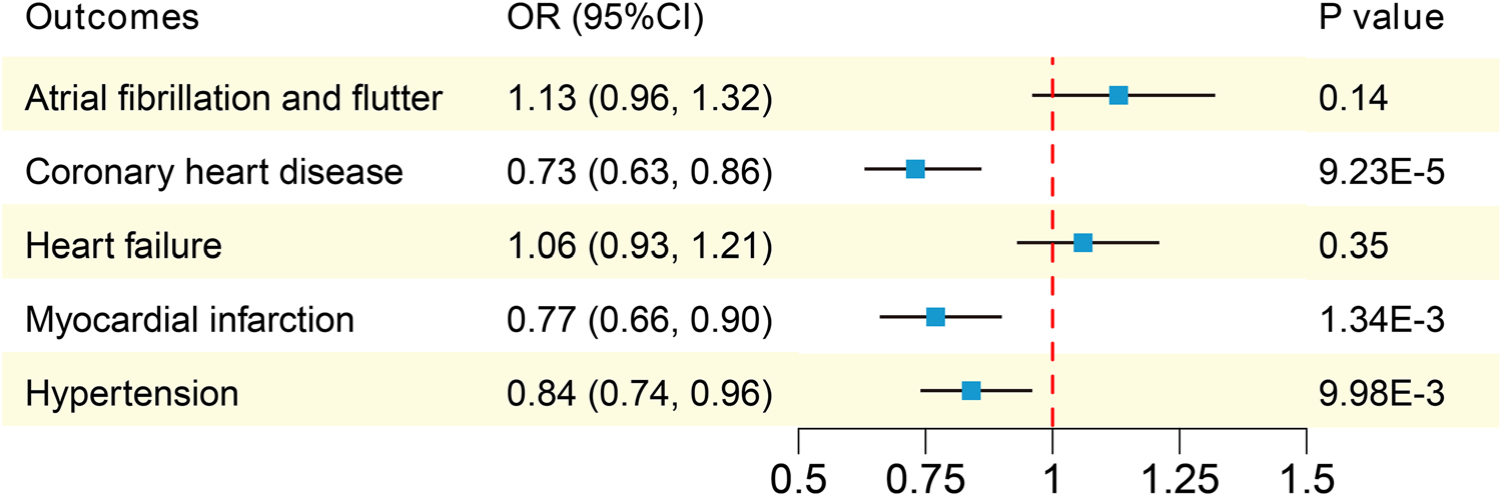
The IVW method to estimate the causal effects of serum SHBG levels on five CVDs. *CVDs* cardiovascular diseases, *IVW* inverse variance weighted, *SHBG* sex hormone-binding globulin, *OR* odds ratio, *CI* confidence interval.

We conducted initial analysis of the causality between SHBG and five CVDs. The IVW method indicates that each increase of one-standard-deviation (1-SD) in SHBG is associated with a reduced risk of CHD (OR 0.73; 95% CI 0.63–0.86), MI (OR 0.77; 95% CI 0.66–0.90), and hypertension (OR 0.84; 95% CI 0.74–0.96), but not with the other two CVDs (Fig. 2). The results of the other three MR analysis methods can be found in Supplementary Fig. S1. The MR-Egger intercept test did not detect horizontal pleiotropy, but all results exhibited varying degrees of heterogeneity (Supplementary Table S26). Since the association between SHBG and CHD is the most significant, we proceeded with the subsequent mediation analysis for this outcome.

### Effects of SHBG on blood lipid levels and CHD

**Figure 3:**
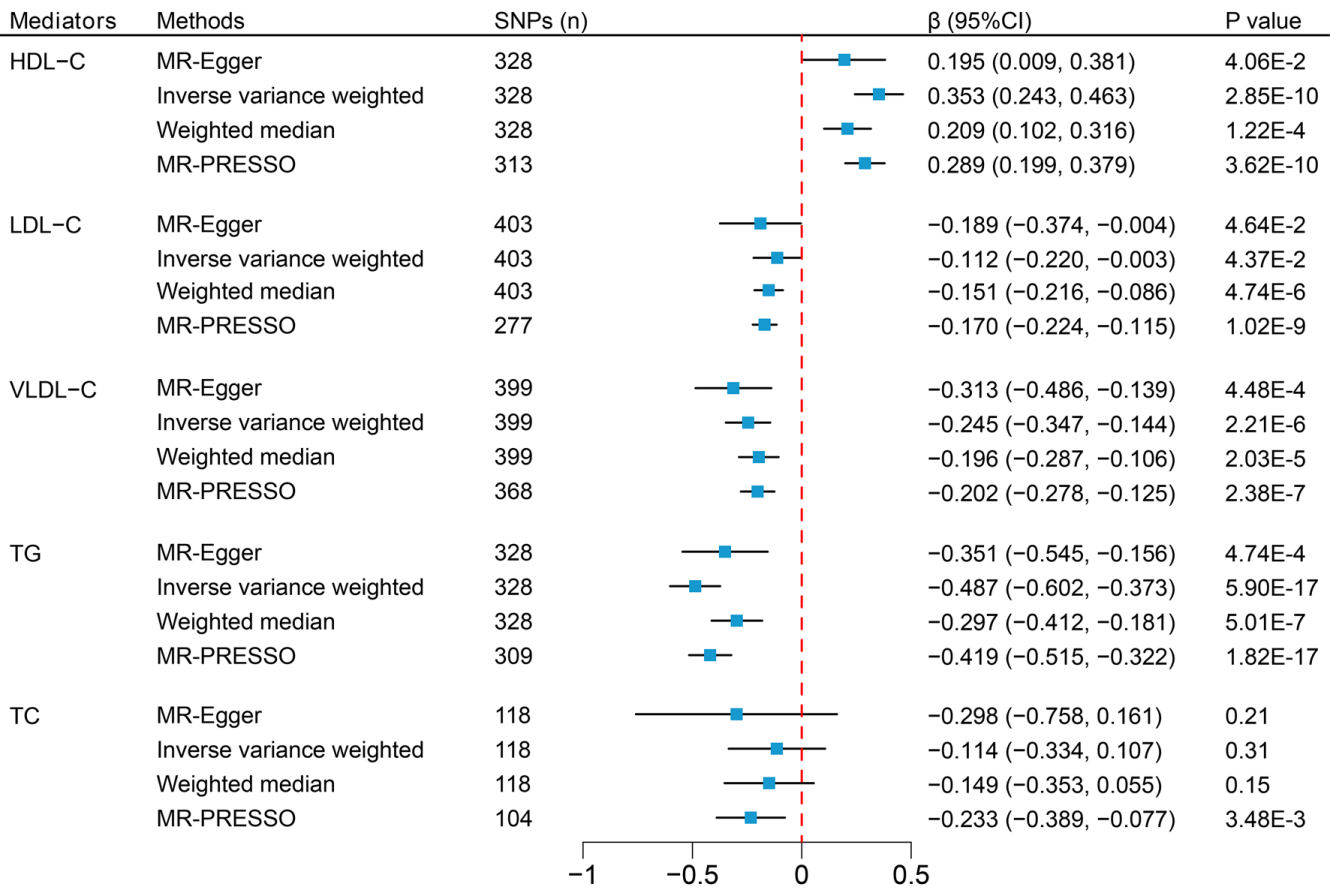
Mendelian randomization analysis to estimate the causal effects of SHBG levels on five continuous phenotypes. *SHBG* sex hormone-binding globulin, *HDL-C* high-density lipoprotein cholesterol, *LDL-C* low-density lipoprotein cholesterol, *VLDL-C* very low-density lipoprotein cholesterol, *TG* triglycerides, *TC* total cholesterol, *SNPs* single-nucleotide polymorphisms, *CI* confidence interval.

**Figure 4:**
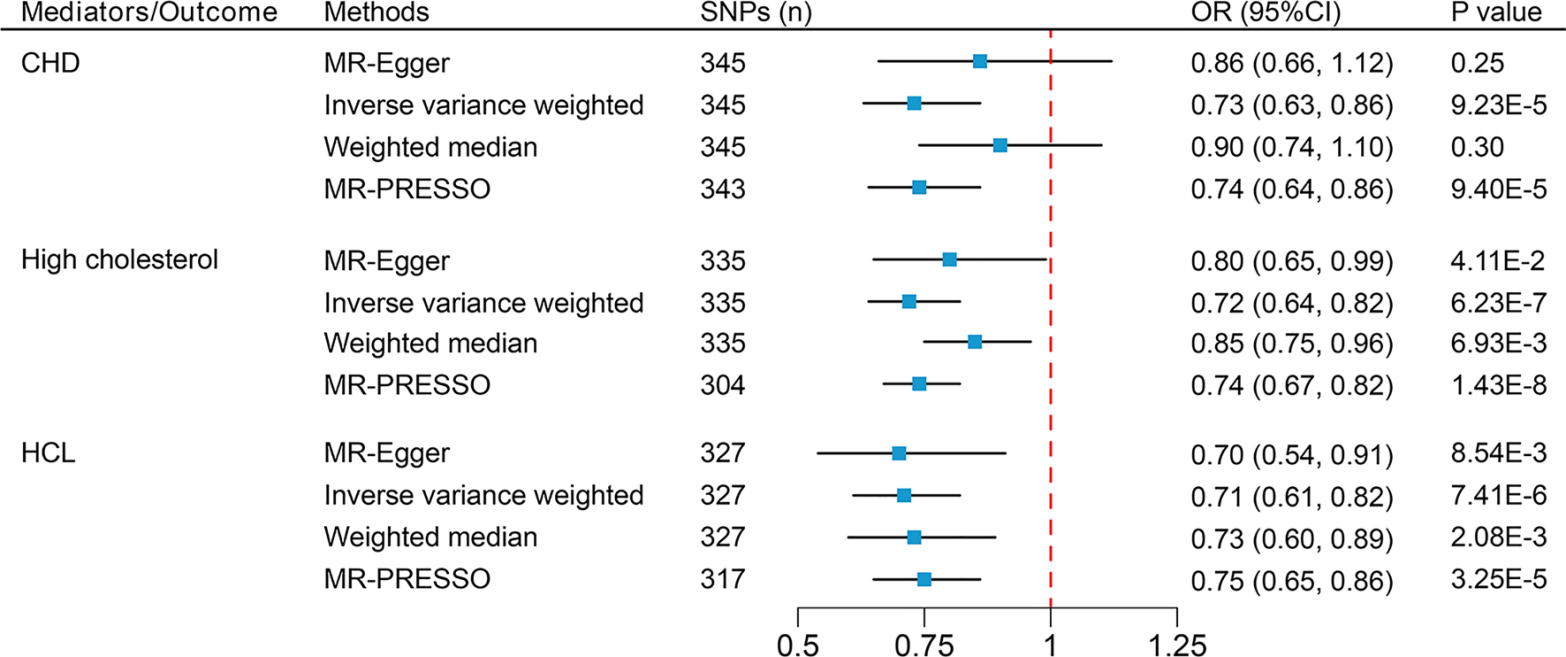
Mendelian randomization analysis to estimate the causal effects of SHBG levels on three dichotomous phenotypes. *SHBG* sex hormone-binding globulin, *CHD* coronary heart disease, *HCL* hypercholesterolemia, *SNPs* single-nucleotide polymorphisms, *OR* odds ratio, *CI* confidence interval.

In the UVMR analysis, the IVW method indicates that each increase of 1-SD in SHBG is associated with a decrease in LDL-C (β = − 0.112; 95% CI − 0.220 to − 0.003), VLDL-C (β = − 0.245; 95% CI − 0.347 to − 0.144), and TG (β = − 0.487; 95% CI − 0.602 to − 0.373) levels, and an increase in HDL-C (β = 0.353; 95% CI 0.243–0.463) levels (Fig. 3). The unit for all effect estimates (i.e., β) is 1-SD. Furthermore, the IVW method also indicates that each increase of 1-SD in SHBG is associated with a reduced risk of high cholesterol (OR 0.72; 95% CI 0.64–0.82), hypercholesterolemia (OR 0.71; 95% CI 0.61–0.82), and CHD (OR 0.73; 95% CI 0.63–0.86) (Fig. 4). The direction of the effect estimates in the other three MR analysis methods is consistent with the IVW method. The MR-Egger intercept test detected horizontal pleiotropy in the MR analysis results of SHBG on HDL-C (intercept = 2.30 × 10^–3^; p-value = 0.04), and all results exhibited varying degrees of heterogeneity (Table 1). However, funnel plots of all results appear relatively symmetric, showing no pronounced heterogeneity or pleiotropy, while scatter plots offer a more intuitive presentation of the results in an alternative format (Supplementary Figs. S2-S9).

**Table 1:** Two sensitivity analyses for the causality between SHBG and mediators/CHD.

Mediators/outcome	Cochran’s Q test		MR-Egger intercept test	
	Q statistic	p-value	Intercept	p-value
HDL-C	1335.05	9.97E−122	2.30E−3	0.04
LDL-C	12,142.96	< 1.00E−300	1.11E−3	0.31
VLDL-C	2244.09	2.47E−254	9.75E−4	0.35
TG	1341.57	8.43E−123	− 1.99E−3	0.09
HCL	1865.36	1.12E−210	− 1.50E−3	0.25
High cholesterol	762.10	6.02E−37	8.20E−5	0.96
TC	733.83	4.99E−90	2.29E−3	0.37
CHD	713.07	5.71E−28	− 2.19E−3	0.17

*SHBG* sex hormone-binding globulin, *CHD* coronary heart disease, *HDL-C* high-density lipoprotein cholesterol, *LDL-C* low-density lipoprotein cholesterol, *VLDL-C* very low-density lipoprotein cholesterol, *TG* triglycerides, *HCL* hypercholesterolemia, *TC* total cholesterol.

The IVW method of the reverse MR analysis indicates a reverse causality between SHBG and CHD, TG, HDL-C, VLDL-C, as well as high cholesterol, but the effect estimates and/or significance of the association are considerably lower than the forward causality (Supplementary Table S27). Corresponding sensitivity analysis reveals significant heterogeneity in all results, and MR-Egger intercept test indicates the presence of horizontal pleiotropy in the reverse MR analysis results between SHBG and HDL-C (intercept = 3.66 × 10^–3^; p-value = 0.02) (Supplementary Table S28). The results of the Steiger directionality test indicate that the direction of the causal effects of SHBG on mediators and CHD is true, and the directionality tests are all significant (p < 0.05).

Therefore, the directionality of the forward causality between SHBG and CHD, as well as all mediators, is established (Table 2). We also used MRlap to correct for the bias caused by sample overlap between SHBG and LDL-C, VLDL-C, high cholesterol, as well as hypercholesterolemia, and there were no significant differences in effect estimates and significance of the association before and after correction (Supplementary Table S29).

**Table 2:** The Steiger directionality test used to validate the validity of the forward causality.

Exposures	Outcomes	SNP r^2^ of exposure	SNP r^2^ of outcome	Correctness of causal direction	p-value of Steiger directionality test
SHBG	High cholesterol	0.122	4.33E−3	True	< 1.00E−300
SHBG	HCL	0.116	1.75E−3	True	< 1.00E−300
SHBG	CHD	0.150	4.72E−3	True	< 1.00E−300
SHBG	HDL-C	0.140	2.46E−2	True	< 1.00E−300
SHBG	LDL-C	0.170	3.21E−2	True	< 1.00E−300
SHBG	VLDL-C	0.170	2.76E−2	True	< 1.00E−300
SHBG	TG	0.140	2.60E−2	True	< 1.00E−300
SHBG	TC	0.050	9.78E−3	True	5.20E−274
HDL-C	CHD	0.098	1.66E−3	True	< 1.00E−300
LDL-C	CHD	0.107	7.88E−3	True	< 1.00E−300
VLDL-C	CHD	0.059	3.20E−3	True	< 1.00E−300
TG	CHD	0.066	1.17E−3	True	< 1.00E−300
High cholesterol	CHD	0.015	3.70E−3	True	5.26E−108
HCL	CHD	2.97E−3	2.93E−3	True	0.878
TC	CHD	0.112	4.04E−3	True	< 1.00E−300

If the causal direction is correct and the directionality test is significant (p < 0.05), it indicates the establishment of forward causality.

*SHBG* sex hormone-binding globulin, *HDL-C* high-density lipoprotein cholesterol, *LDL-C* low-density lipoprotein cholesterol, *VLDL-C* very low-density lipoprotein cholesterol, *TG* triglycerides, *HCL* hypercholesterolemia, *TC* total cholesterol, *CHD* coronary heart disease, *SNP* single nucleotide polymorphism.

Taking into account the results from the above analysis, based on the previously established definition of significant causality, the causality between SHBG and CHD, high cholesterol, hypercholesterolemia, LDL-C, VLDL-C, as well as TG are established.

### Effects of blood lipid levels on CHD

In the UVMR analysis of mediators and CHD, the IVW method indicates that each increase of 1-SD in LDL-C, VLDL-C, TG, and TC is associated with an increased risk of CHD. High cholesterol and hypercholesterolemia are also associated with an increased risk of CHD, while each increase of 1-SD in HDL-C is associated with a reduced risk of CHD (Supplementary Fig. S10). The MR-Egger intercept test detected horizontal pleiotropy in the MR analysis results of TC on CHD, and all results exhibited varying degrees of heterogeneity (Supplementary Table S30). All funnel plots and scatter plots for the MR analyses can be found in Supplementary Fig. S11–S17. For some of the analysis results, we also constructed forest plots of individual SNPs and conducted leave-one-out analysis (Supplementary Fig. S15–S17).

The IVW method of the reverse MR analysis indicates a reverse causality between CHD and LDL-C, high cholesterol, as well as hypercholesterolemia (Supplementary Table S27). Corresponding sensitivity analysis reveals significant heterogeneity in all results, and MR-Egger intercept test did not detect horizontal pleiotropy (Supplementary Table S28). The results of the Steiger directionality test indicate that the direction of the causal effects of mediators on CHD is true, and most directionality tests are significant (p < 0.05) except for the result of hypercholesterolemia (p = 0.878). Therefore, except for hypercholesterolemia, the directionality of the forward causality between mediators and CHD is established (Table 2).

Similarly, based on our established definition of significant causality, the causality between the 6 mediators (high cholesterol, HDL-C, LDL-C, VLDL-C, TG, as well as TC) and CHD are established.

### Mediation by blood lipid levels

**Figure 5:**
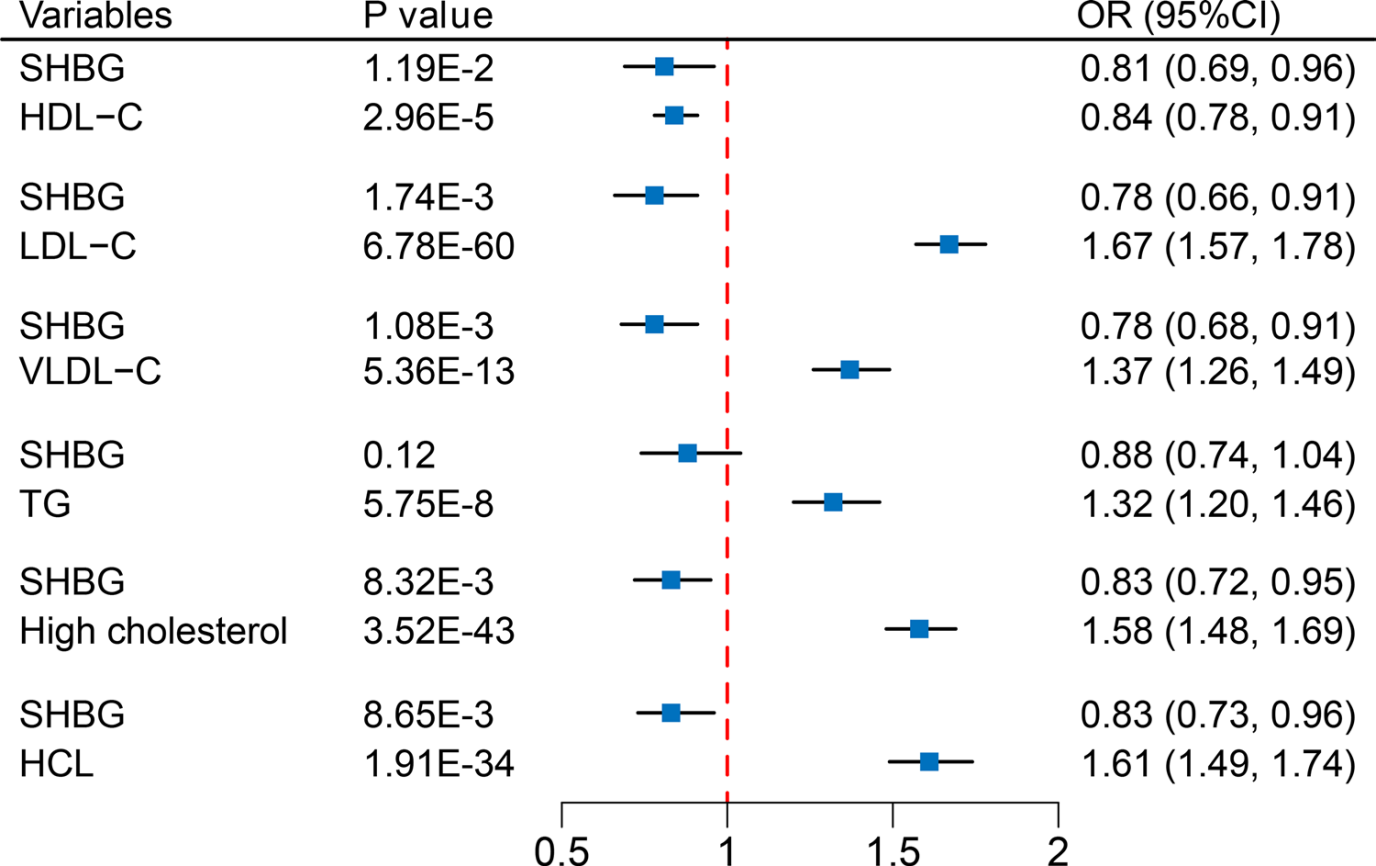
Multivariable Mendelian randomization to assess the direct effects on CHD. Multivariable Mendelian randomization involves mutual adjustment for serum SHBG levels and blood lipid levels. *SHBG* sex hormone-binding globulin, *CHD* coronary heart disease, *HDL-C* high-density lipoprotein cholesterol, *LDL-C* low-density lipoprotein cholesterol, *VLDL-C* very low-density lipoprotein cholesterol, *TG* triglycerides, *HCL* hypercholesterolemia, *OR* odds ratio, *CI* confidence interval.

The MVMR analysis provided direct effects of SHBG and blood lipid levels on CHD. Even after adjusting for SHBG, the causality between all mediators and CHD persists. Every increase of 1-SD in LDL-C (OR 1.67; 95% CI 1.57–1.78), VLDL-C (OR 1.37; 95% CI 1.26–1.49), and TG (OR 1.32; 95% CI 1.20–1.46) is associated with an increased risk of CHD. High cholesterol (OR 1.58; 95% CI 1.48–1.69) and hypercholesterolemia (OR 1.61; 95% CI 1.49–1.74) are also associated with an increased risk of CHD, while each increase of 1-SD in HDL-C (OR 0.84; 95% CI 0.78–0.91) is associated with a reduced risk of CHD (Fig. 5). Except for hypercholesterolemia (F-statistic = 3.8), all variables in the MVMR analysis exhibit sufficient IV strength; all MVMR analysis results exhibit varying degrees of heterogeneity (Supplementary Table S31).

**Figure 6:**
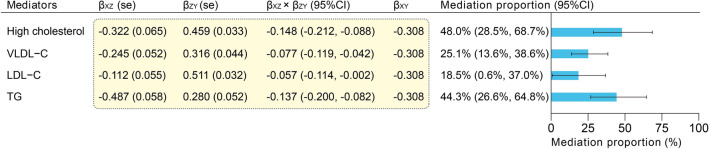
Mediating effect of blood lipid levels in the causality between SHBG and CHD. 95% CI for the mediation proportion calculated using the delta method. *SHBG* sex hormone-binding globulin, *CHD* coronary heart disease, *CI* confidence interval, *VLDL-C* very low-density lipoprotein cholesterol, *LDL-C* low-density lipoprotein cholesterol, *TG* triglycerides, *se* standard error.

We conducted a mediation analysis for the causality between SHBG and CHD using the effect estimates obtained. The delta method was the main approach for calculating 95% CI of mediation proportion. The mediation proportions for high cholesterol (48.0%; [95% CI 28.5–68.7%]), VLDL-C (25.1%; [95% CI 13.6–38.6%]), LDL-C (18.5%; [95% CI 0.6–37.0%]), and TG (44.3%; [95% CI 26.6–64.8%), as well as the necessary effect estimates, are shown in Fig. 6. Additionally, we also calculated 95% CI of mediation proportion using the Bootstrap method (Supplementary Fig. S18). The mediation proportion calculated by the difference method can be found in Supplementary Fig. S19.

## The sex-specific causality between SHBG and the risk of CHD

**Figure 7:**
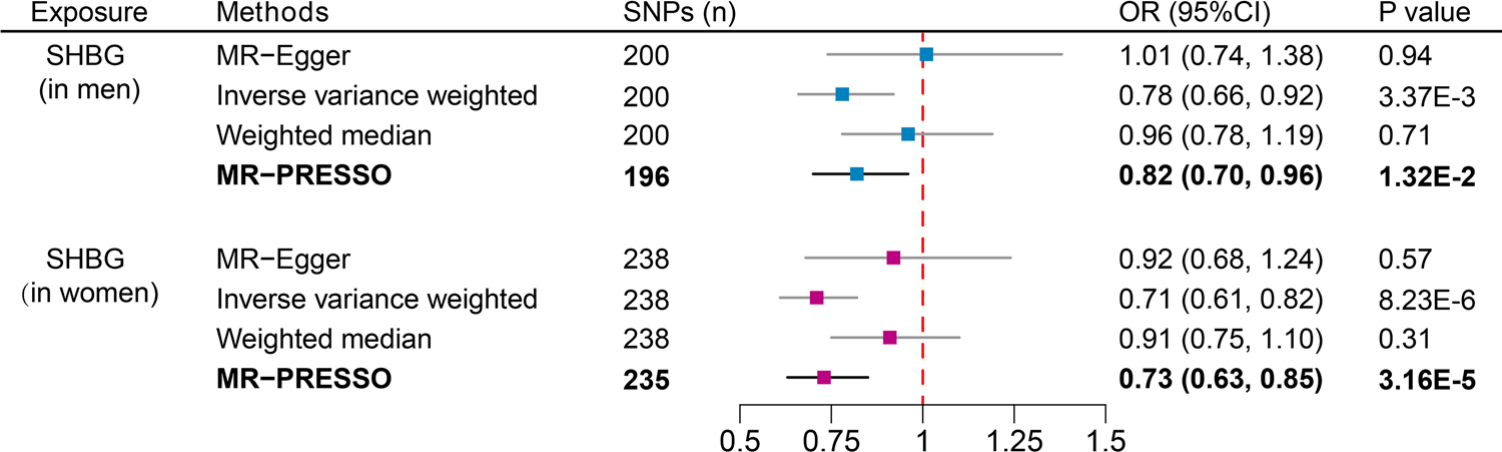
Sex-specific analysis to explore the sex-specific causal effects of serum SHBG levels on CHD. *SHBG* sex hormone-binding globulin, *CHD* coronary heart disease, *SNPs* single-nucleotide polymorphisms, *OR* odds ratio, *CI* confidence interval.

The IVW method reveals that with each increase of 1-SD in SHBG, the reduction in CHD risk is greater in the females (OR 0.71; 95% CI 0.61–0.82; p-value = 8.23 × 10^–6^) compared to the males (OR 0.78; 95% CI 0.66–0.92; p-value = 3.37 × 10^–3^), and the significance of this association is also greater in females than in males (Fig. 7). However, the MR analysis results exhibit pleiotropy and heterogeneity (Supplementary Table S32). The results from MR-PRESSO with outliers removed may be relatively more accurate, but overall, the obtained results are considered to be unreliable.

## Discussion

This MR study demonstrates negative causality between genetically predicted serum SHBG levels and the risk of CHD, MI, as well as hypertension. We found that the causal effect of reducing CHD risk is largely achieved through improving lipid profiles, and we have identified the proportions mediated by blood VLDL-C, LDL-C, TG levels, and high cholesterol.

A meta-analysis incorporating ten previous observational studies indicates that circulating SHBG levels are associated with lower CHD risk in both men and women^10^. Furthermore, the results of two recent MR studies on the same topic also support the conclusion that an increase in SHBG levels is associated with a reduced risk of CHD^10,44^. Our mediation analysis highlights that this biological effect of SHBG is largely achieved by improving the blood lipid profiles.

As early as the 1990s, studies had already revealed the association between SHBG and blood lipid levels, primarily limited to the finding of an inverse correlation between SHBG and HDL-C, without elucidating its relationship with other lipid components^45,46^. Two subsequent cross-sectional studies involving healthy men indicated that low SHBG is associated with an atherogenic lipid profile (low HDL, high VLDL, and high TG); higher SHBG concentrations are related to lower TC, TG, and higher HDL-C^8,47^. However, given the substantial differences in reproductive endocrinology between males and females, the levels and physiological effects of SHBG may be different between the two groups, and the gender limitation makes it difficult to generalize the conclusions of this study to the entire population. However, a recent cross-sectional study involving 3231 postmenopausal women reported similar results. The serum SHBG levels in the second to fourth quartiles were positively correlated with HDL-C and inversely correlated with non-HDL-C and TG^6^. Furthermore, a large cohort study conducted by Elif et al., involving 3264 men and women, also indicated an inverse correlation between serum TG and SHBG^7^. The accumulating body of related research has progressively substantiated the role of SHBG in lipid improvement, with the evidence regarding the correlation between SHBG and HDL-C being the most substantial. Unfortunately, our findings in this regard are subject to horizontal pleiotropy, making it impossible to establish a definitive relationship between them.

The earliest known physiological function of SHBG is to bind to sex hormones, thereby regulating their bioavailability^48^. The activity of hepatic lipase involved in HDL-C breakdown is stimulated by androgens, while high levels of SHBG can reduce free androgens, thus decreasing hepatic lipase activity and increasing HDL-C levels^49^. Furthermore, previous studies indicate that the interaction between AMP-activated protein kinase and peroxisome proliferator-activated receptor can regulate the expression of hepatic nuclear factor-4α, thereby upregulating SHBG expression. Moreover, hepatic nuclear factor-4α influences the transcription of numerous genes related to lipid metabolism, which may help explain the correlation between circulating SHBG levels and lipid metabolism^50^. In summary, while epidemiology has revealed an association between SHBG and blood lipids, the underlying biological mechanisms remain poorly understood. As for dyslipidemia being a significant risk factor for CHD and its involvement in the pathophysiology of atherosclerosis, these have already been confirmed by previous studies^51,52^. Low-density lipoprotein is considered the most important blood lipid component leading to atherosclerosis, which is consistent with our findings^13^. Currently, clinical trials have established statin as the cornerstone of lipid-lowering therapy for CHD^53^. Good adherence to lipid-lowering therapy and achievement of LDL-C control target can significantly reduce the occurrence of major adverse cardiovascular events in patients with CHD^54^. Of course, besides alterations in lipid levels, there are inevitably other mediators at play. Studies indicate that low SHBG is also associated with insulin resistance, obesity, and other manifestations of metabolic syndrome^50^, all of which contribute to the development of atherosclerosis^55,56^.

In current clinical practice, serum SHBG is generally considered to have significant diagnostic value in polycystic ovary syndrome, while in other situations, SHBG measurement is rarely performed^57,58^. The increased risk of dyslipidemia and CHD associated with low SHBG levels may be largely overlooked. Therefore, our findings can assist clinical investigators in reevaluating the significance and necessity of detecting this parameter. As for whether SHBG can serve as a crucial marker for assessing the occurrence of related diseases, further research is needed to explore this possibility. In addition, it is important to note that the phenotypes predicted by the genetic variations begins early in life, thereby providing an estimate of lifelong effects, which differs from traditional clinical studies that apply interventions or observe phenotypes only for a period of time^20,59^. This is particularly crucial for the causality revealed in this study, as it can be ascertained that the influence of lipid levels on CHD risk is a long-term cumulative effect^20,59^. Because MR reveals long-term effects, short-term interventions targeting SHBG and lipid levels may yield effects lower than the expected effect estimates obtained from this MR study^60^.

There is a significant gender disparity in the risk of CHD, with women having a much lower risk of new-onset coronary events and related all-cause mortality. However, this "gender protection effect" significantly diminishes following MI^61^. In fact, women have unique atypical risk factors that are associated with the prognosis of CVDs, such as pre-menopausal breast fat accumulation, which negatively affect cardiovascular function through the overexpression of sodium-glucose transporter 2 and inflammatory cytokines downregulating the breast sirtuins^62,63^. This suggests that the influence of gender on the pathogenesis of CVDs may be quite complex. Benefiting from the SHBG summary-level data stratified by gender, we attempted a sex-specific analysis. Unfortunately, due mainly to the "emerging" horizontal pleiotropy, we cannot conclude that there is a sex difference in the effect of SHBG on CHD risk. Considering the principle behind horizontal pleiotropy, it's possible that when conducting MR analysis using SHBG summary-level data from male and female samples separately, the effects of certain confounders be amplified or new confounders arise.

The main strength of our study is that we utilized large GWAS summary-level data to comprehensively investigate the interrelationships between SHBG, lipid profiles, and CHD. This is the first MR study to provide causal evidence that blood lipid levels mediated a considerable proportion of serum SHBG effect on CHD risk, and extensive sensitivity analyses were conducted to assess the robustness of the results. This study also has some limitations, and we have made extensive efforts to address them. **First**, the IVW method of bidirectional MR shows reverse causality between many phenotypes, but the effect size and/or significance levels are much smaller than those observed for forward causality. Furthermore, the Steiger directionality test validates the effectiveness of the vast majority of forward causality. Because the methodology of MR is inherently less susceptible to the spurious reverse causality, bidirectional MR can indeed suggest that two phenotypes drive each other, and this situation does not affect the validity of forward causality^64^. Second, there is mild horizontal pleiotropy in the MR analysis results for SHBG and HDL-C (p = 0.02), which could potentially violate assumption II and III, leading to inaccurate causal estimates^40^. Therefore, we did not establish the association between them. Third, there is sample overlap between SHBG and partial mediators. Nevertheless, one study suggests that 2-sample MR methods can be safely used for 1-sample MR when the sample size is large^65^. In addition, the minimal discrepancy observed in the results before and after MRlap correction could also suggests that sample overlap is unlikely to have a significant impact on the causal effect. Lastly, the difference in the mediation proportions calculated by the difference method and the product method is negligible, further indicating that the impact caused by sample overlap is minimal. Fourth, in the GWAS for CHD and MI, 23% of the participants are of non-European ancestry. Population stratification may introduce confounding of the relationship between genetic variants and outcome, thus posing a risk of violating assumption III^66^. However, we observed that the effect allele frequency of the GWAS sample population for these two outcomes and serum SHBG levels are essentially consistent. This suggests that the racial heterogeneity is minor, and it is unlikely to introduce perceptible bias. Fifth, to some extent, due to the large number of SNPs, almost all MR analyses exhibit considerable heterogeneity. Therefore, the IVW method uniformly employs random-effects model. Sixth, the GWAS involved in our MR study was conducted in European population, so our findings may be difficult to generalize to other ethnic groups.

## Conclusion

High serum SHBG levels are causally associated with reduced risk of CHD, MI, and hypertension, in which the improvement of lipid profile largely mediates the causal effect of CHD risk reduction. Our study results further emphasize the close association between SHBG and lipid metabolism, which subsequently impacts the onset of CHD. However, due to some limitations, our findings still need to be further explored and confirmed by future studies.

### Supplementary Information

Supplementary Tables.

Supplementary Information.

## Data Availability

The summary-level data for high cholesterol and hypercholesterolemia is sourced from the Bristol University Data Repository (https://data.bris.ac.uk/data/dataset/pnoat8cxo0u52p6ynfaekeigi). For specific instructions on how to request and download the data, refer to the MRC IEU UK Biobank GWAS pipeline version 2^28^. The summary-level data for SHBG can be obtained from the UK Biobank (https://www.ukbibank.ac.uk). The summary-level data for VLDL-C also originates from the UK Biobank resource (https://biobank.ndph.ox.ac.uk/showcase/label.cgi?id=220). The summary-level data for TC^23^, CHD^29^, and MI^29^ are sourced from publicly published GWAS. The summary-level data for TG, LDL-C, and HDL-C were obtained from the IEU OpenGWAS project (https://gwas.mrcieu.ac.uk/datasets). The summary-level data for hypertension, heart failure and atrial fibrillation & flutter originate from the FinnGen Consortium (https://finngen.gitbook.io/documentation/). Additionally, the summary-level data for SHBG, TC, LDL-C, and CHD can also be found in the GWAS Catalog (https://www.ebi.ac.uk/gwas/home). Datasets generated during the current study are included in the Supplementary Material, further inquiries can be directed to the corresponding authors.
