# Supplementary material for: Blood lipid levels mediating the effects of sex hormone-binding globulin on coronary heart disease: Mendelian randomization and mediation analysis
Source: Sci Rep. 2024 May 25;14:11993. doi: 10.1038/s41598-024-62695-8 (PMC11127952; doi:10.1038/s41598-024-62695-8)
Supplement: Supplementary file 2 — Supplementary Information. [file 41598_2024_62695_MOESM2_ESM.pdf]

Supplementary Material

Blood Lipid Levels Mediating the Effects of Sex Hormone-Binding Globulin on Coronary Heart Disease: Mendelian Randomization and Mediation Analysis

Author list: Juntao Yang, Jiedong Zhou, Hanxuan Liu, Jinjin Hao, Songqing Hu, Peipei Zhang, Haowei Wu, Yefei Gao, Weiliang Tang

Corresponding author: Weiliang Tang. Department of Cardiology, Shaoxing People's Hospital, Shaoxing, Zhejiang 312000, P. R. China; E-mail: twl-sxyz@163.com

Contents

Supplementary Figure S1. Other three Mendelian randomization analysis methods used to study the association between SHBG and five CVDs 5

Supplementary Figure S2. Scatter plot and funnel plot for the association of SHBG with CHD 5

Supplementary Figure S3. Scatter plot and funnel plot for the association of SHBG with HDL-C 6

Supplementary Figure S4. Scatter plot and funnel plot for the association of SHBG with LDL-C 6

Supplementary Figure S5. Scatter plot and funnel plot for the association of SHBG with VLDL-C 7

Supplementary Figure S6. Scatter plot and funnel plot for the association of SHBG with TG 7

Supplementary Figure S7. Scatter plot and funnel plot for the association of SHBG with high cholesterol 8

Supplementary Figure S8. Scatter plot and funnel plot for the association of SHBG with HCL. 8

Supplementary Figure S9. Scatter plot and funnel plot for the association of SHBG with TC 9

Supplementary Figure S11. Scatter plot and funnel plot for the association of HDL-C with CHD 11

Supplementary Figure S12. Scatter plot and funnel plot for the association of LDL-C with CHD 11

Supplementary Figure S13. Scatter plot and funnel plot for the association of high cholesterol with CHD 12

Supplementary Figure S14. Scatter plot and funnel plot for the association of TC with CHD 12

Supplementary Figure S15. Scatter plot, funnel plot, leave-one-out sensitivity analysis and forest plot of a single SNP for the association of VLDL-C with CHD 13

Supplementary Figure S16. Scatter plot, funnel plot, leave-one-out sensitivity analysis and forest plot of a single SNP for the association of TG with CHD 14

Supplementary Figure S17. Scatter plot, funnel plot, leave-one-out sensitivity analysis and forest plot of a single SNP for the association of HCL with CHD 15

Supplementary Figure S18. Mediating effect of blood lipid level in the causality between SHBG and CHD (95% CI for the mediation proportion calculated using the Bootstrap method) 15

Supplementary Figure S19. Mediating effect of partial mediators that is calculated by difference method in the causality between SHBG and CHD (with GWAS sample overlap between the SHBG and mediators) 16

Supplementary Table S1. Detailed information on GWAS summary-level data of the continuous phenotypes 17

Supplementary Table S2. Detailed information on GWAS summary-level data of the dichotomous phenotypes 17

Supplementary Table S3. Characteristics of GWAS summary-level data for continuous phenotypes 18

Supplementary Table S4. Diagnosis and definition of dichotomous phenotypes (diseases) 19

Supplementary Table S5. Instrumental variables used for the Mendelian randomization analysis of the effect of serum SHBG levels on CHD risk 19

Supplementary Table S6. Instrumental variables used for the Mendelian randomization analysis of the effect of serum SHBG levels on high cholesterol risk 19

Supplementary Table S7. Instrumental variables used for the Mendelian randomization analysis of the effect of serum SHBG levels on hypercholesterolemia risk 19

Supplementary Table S8. Instrumental variables used for the Mendelian randomization analysis of the effect of serum SHBG levels on VLDL-C levels 20

Supplementary Table S9. Instrumental variables used for the Mendelian randomization analysis of the effect of serum SHBG levels on LDL-C levels 20

Supplementary Table S10. Instrumental variables used for the Mendelian randomization analysis of the effect of serum SHBG levels on triglycerides levels 20

Supplementary Table S11. Instrumental variables used for the Mendelian randomization analysis of the effect of serum SHBG levels on HDL-C levels 20

Supplementary Table S12. Instrumental variables used for the Mendelian randomization analysis of the effect of serum SHBG levels on total cholesterol levels 20

Supplementary Table S13. Instrumental variables used for the Mendelian randomization analysis of the effect of high cholesterol on CHD risk 20

Supplementary Table S14. Instrumental variables used for the Mendelian randomization analysis of the effect of hypercholesterolemia on CHD risk 20

Supplementary Table S15. Instrumental variables used for the Mendelian randomization analysis of the effect of VLDL-C levels on CHD risk 21

Supplementary Table S16. Instrumental variables used for the Mendelian randomization analysis of the effect of LDL-C levels on CHD risk 21

Supplementary Table S17. Instrumental variables used for the Mendelian randomization analysis of the effect of triglycerides levels on CHD risk 21

Supplementary Table S18. Instrumental variables used for the Mendelian randomization analysis of the effect of HDL-C levels on CHD risk 21

Supplementary Table S19. Instrumental variables used for the Mendelian randomization analysis of the effect of total cholesterol levels on CHD risk 21

Supplementary Table S20. Instrumental variables used for the multivariable Mendelian randomization analysis of the effect of serum SHBG levels and high cholesterol on CHD risk 21

Supplementary Table S21. Instrumental variables used for the multivariable Mendelian randomization analysis of the effect of serum SHBG levels and hypercholesterolemia on CHD risk 21

Supplementary Table S22. Instrumental variables used for the multivariable Mendelian randomization analysis of the effect of serum SHBG and VLDL-C levels on CHD risk 22

Supplementary Table S23. Instrumental variables used for the multivariable Mendelian randomization analysis of the effect of serum SHBG and LDL-C levels on CHD risk 22

Supplementary Table S24. Instrumental variables used for the multivariable Mendelian randomization analysis of the effect of serum SHBG and triglycerides levels on CHD risk 22

Supplementary Table S25. Instrumental variables used for the multivariable Mendelian randomization analysis of the effect of serum SHBG and HDL-C levels on CHD risk 22

Supplementary Table S26. Two sensitivity analyses for the causality between SHBG and five CVDs 22

Supplementary Table S27. Four Mendelian randomization analysis methods used to study the reverse causality between SHBG, mediators and CHD pairwise 23

Supplementary Table S28. Two sensitivity analyses for the reverse causality between SHBG, mediators and CHD pairwise 25

Supplementary Table S29. Effect estimates of the causality between SHBG and four mediators (corrected for bias primarily induced by sample overlap using MRlap) 25

Supplementary Table S30. Two sensitivity analyses for the causality between mediators and CHD 26

Supplementary Table S31. F-statistics calculation and Cochran's Q test for MVMR analyses 26

Supplementary Table S32. Two sensitivity analyses for sex-specific causality between SHBG and CHD 27

Supplementary Table S33. STROBE-MR checklist of recommended items to address in reports of Mendelian randomization study 27

Supplementary Methods S1. Detailed information about the database involved in this study 30

Supplementary Methods S2. Describing how to handle GWAS summary-level data of variables 31

Supplementary Methods S3. MRlap introduction and method description 31

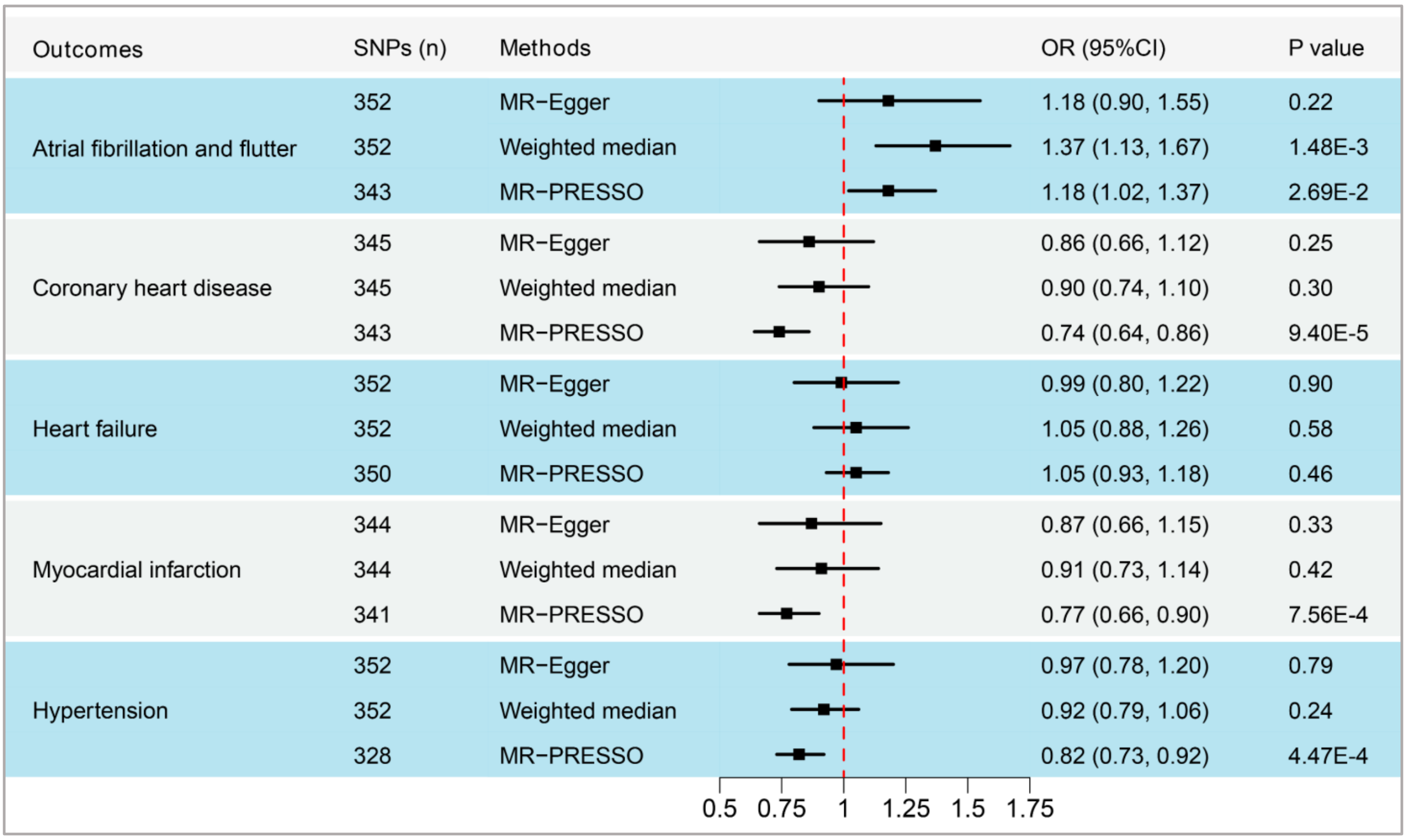

Supplementary Figure S1. Other three Mendelian randomization analysis methods used to study the association between SHBG and five CVDs. SHBG, sex hormone-binding globulin; CVDs, cardiovascular diseases; SNPs, single nucleotide polymorphisms; OR, odds ratio; CI, confidence interval.

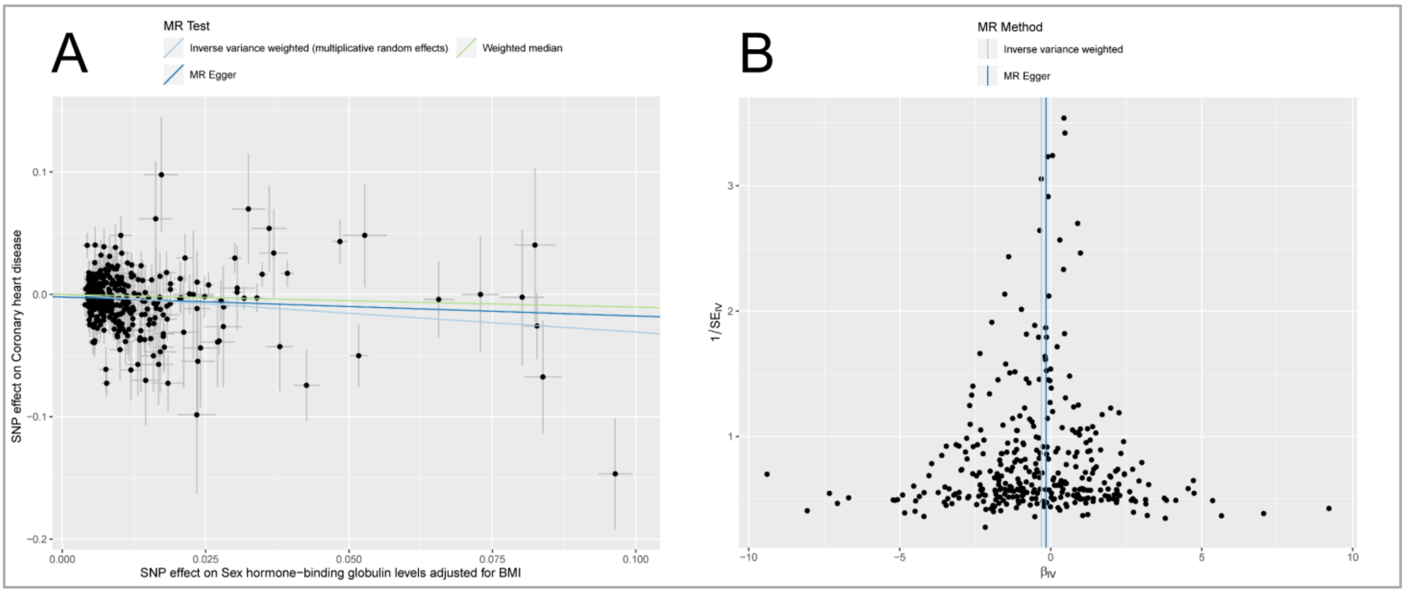

Supplementary Figure S2. Scatter plot and funnel plot for the association of SHBG with CHD. SHBG, sex hormone-binding globulin; CHD, coronary heart disease; SNP, single nucleotide polymorphism; SE, standard error; IV, instrumental variable; BMI, body mass index.

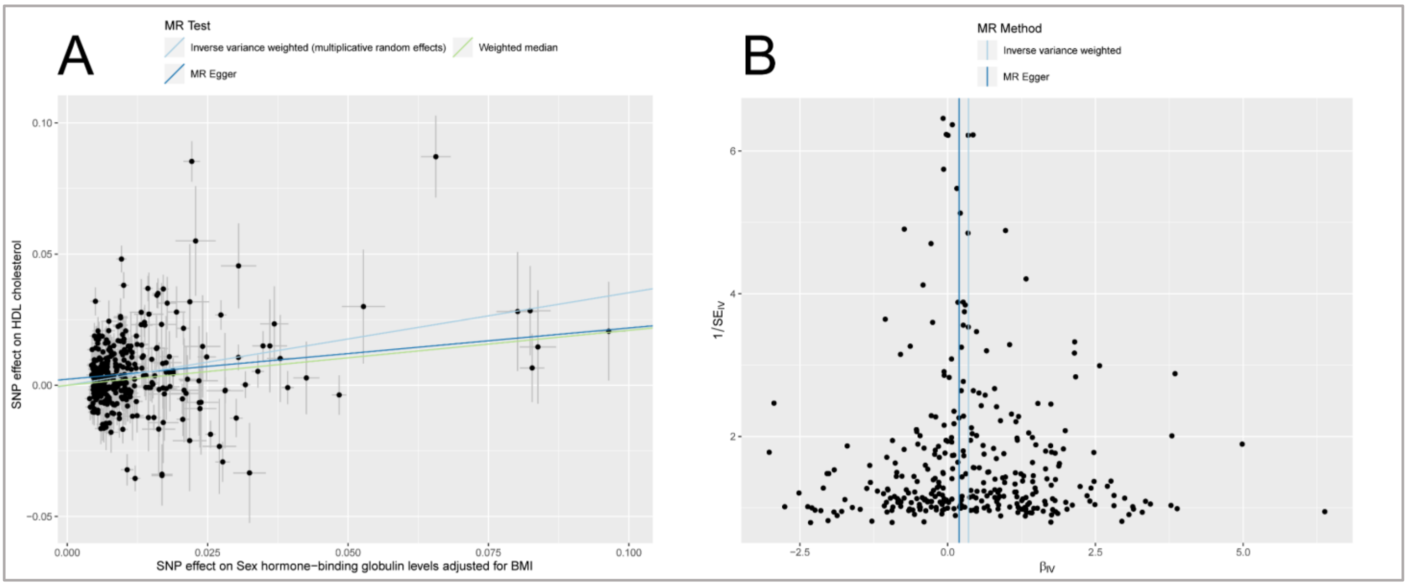

Supplementary Figure S3. Scatter plot and funnel plot for the association of SHBG with HDL-C. SHBG, sex hormone-binding globulin; HDL-C, high-density lipoprotein cholesterol; SNP, single nucleotide polymorphism; SE, standard error; IV, instrumental variable; BMI, body mass index.

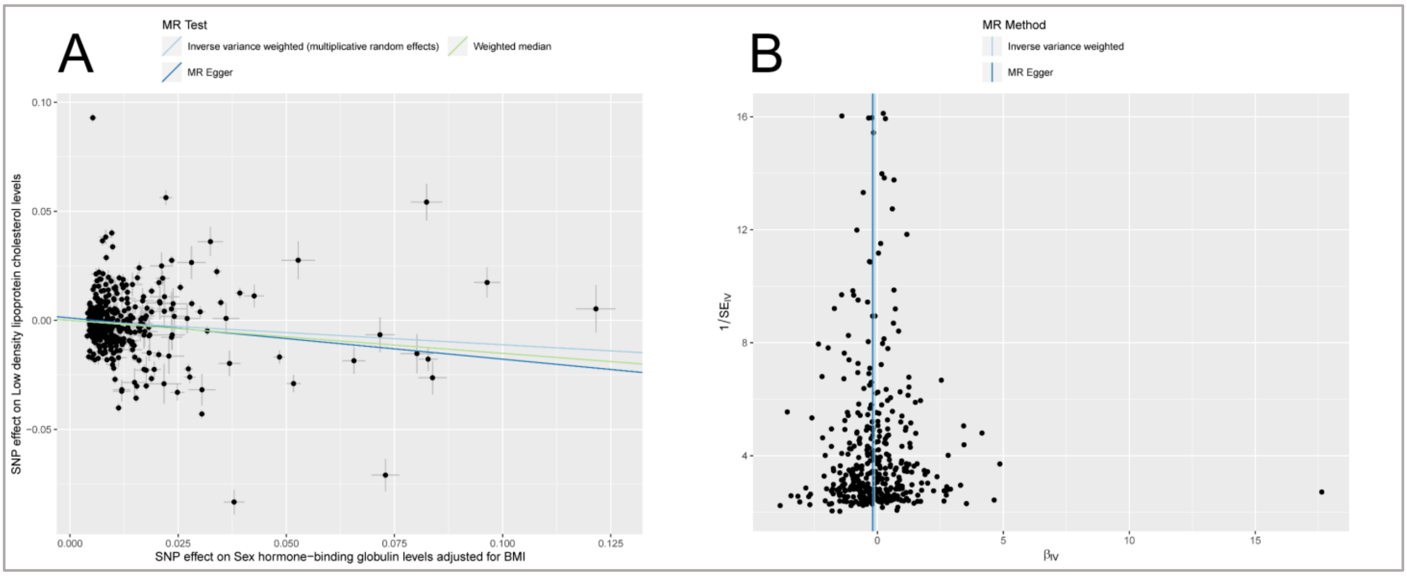

Supplementary Figure S4. Scatter plot and funnel plot for the association of SHBG with LDL-C. SHBG, sex hormone-binding globulin; LDL-C, low-density lipoprotein cholesterol; SNP, single nucleotide polymorphism; SE, standard error; IV, instrumental variable; BMI, body mass index.

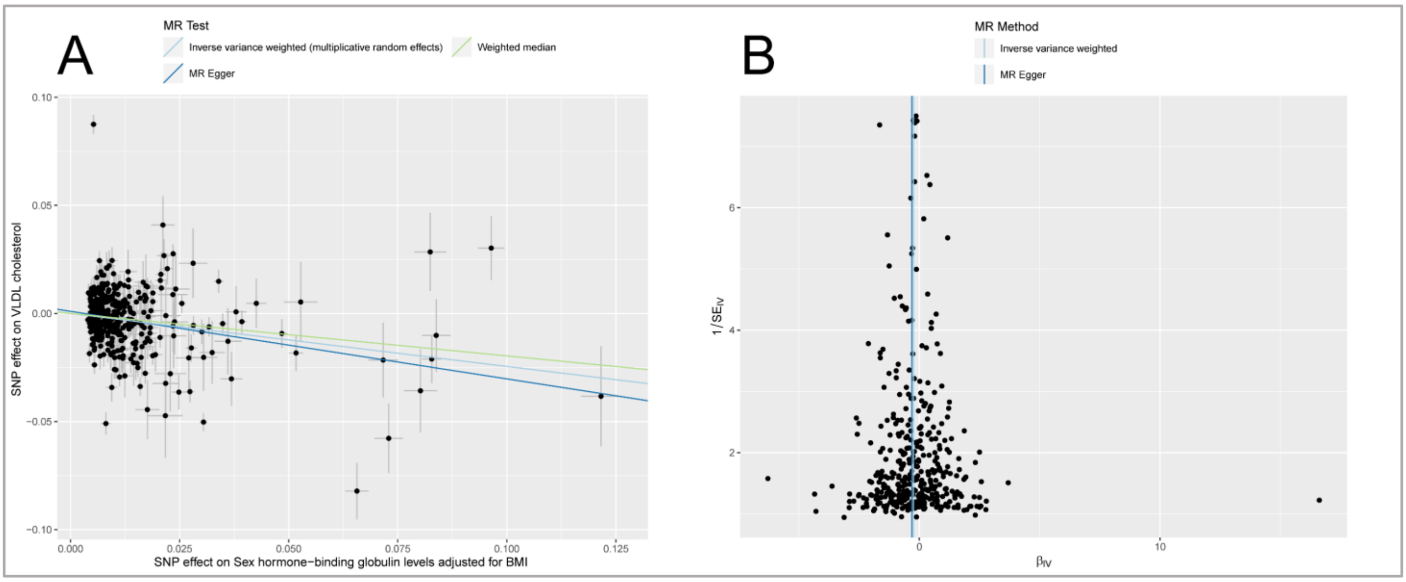

Supplementary Figure S5. Scatter plot and funnel plot for the association of SHBG with VLDL-C. SHBG, sex hormone-binding globulin; VLDL-C, very low-density lipoprotein cholesterol; SNP, single nucleotide polymorphism; SE, standard error; IV, instrumental variable; BMI, body mass index.

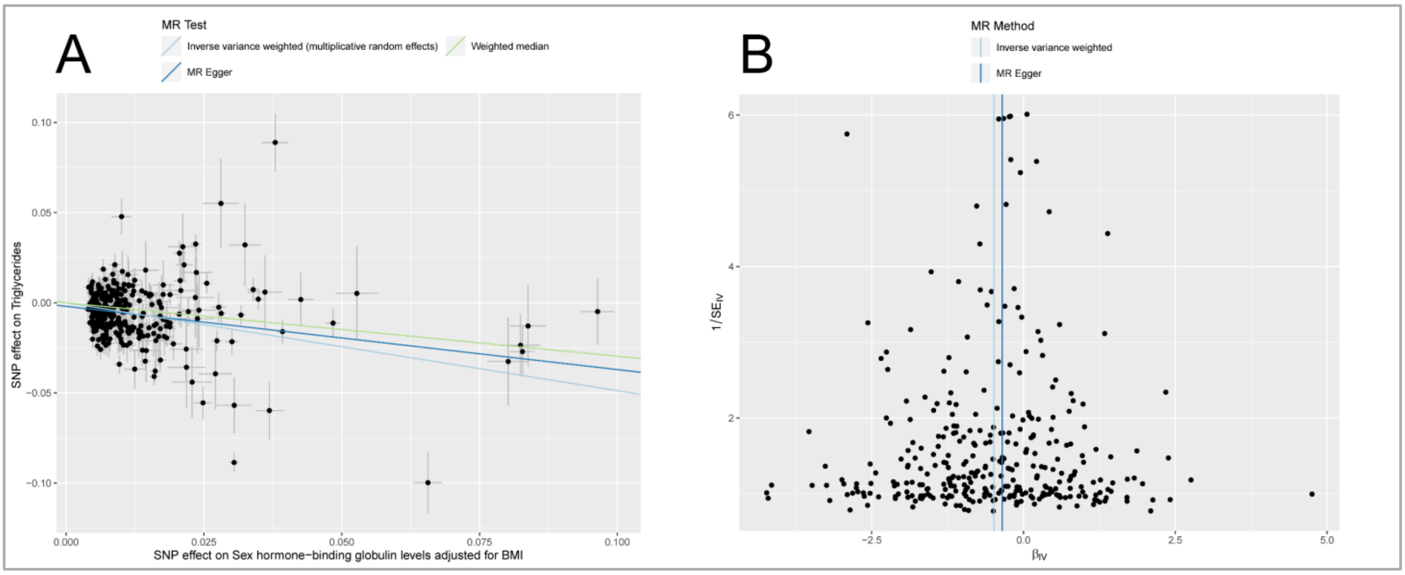

Supplementary Figure S6. Scatter plot and funnel plot for the association of SHBG with TG. SHBG, sex hormone-binding globulin; TG, triglycerides; SNP, single nucleotide polymorphism; SE, standard error; IV, instrumental variable; BMI, body mass index.

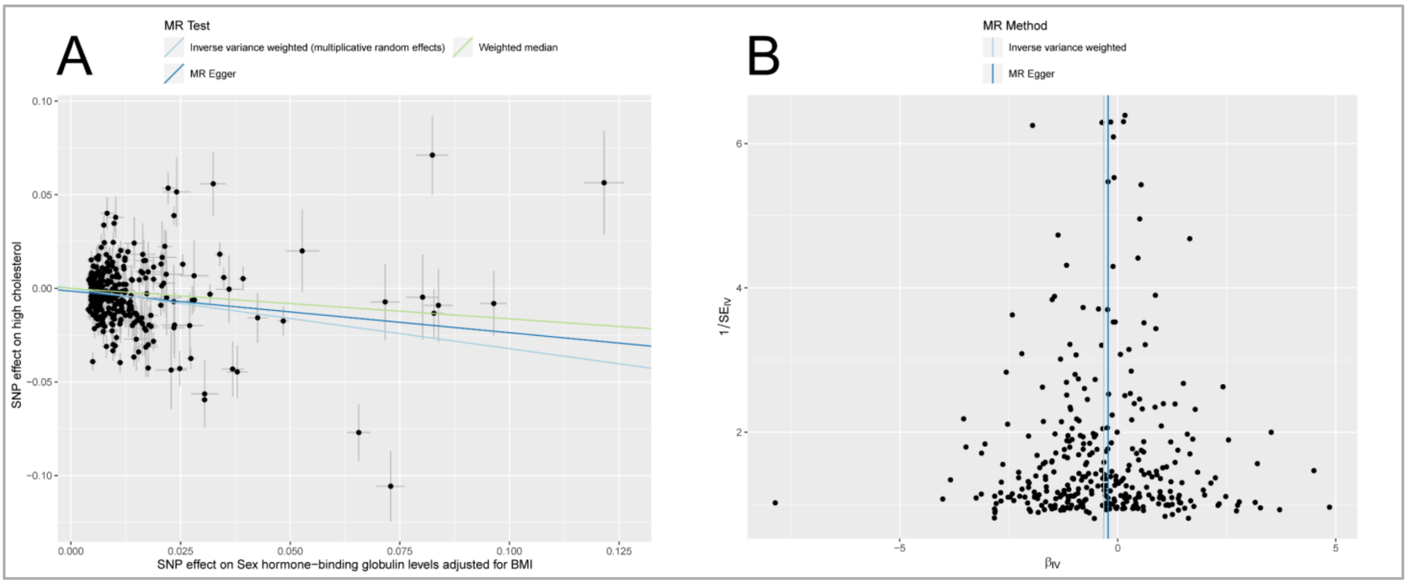

Supplementary Figure S7. Scatter plot and funnel plot for the association of SHBG with high cholesterol. SHBG, sex hormone-binding globulin; SNP, single nucleotide polymorphism; SE, standard error; IV, instrumental variable; BMI, body mass index.

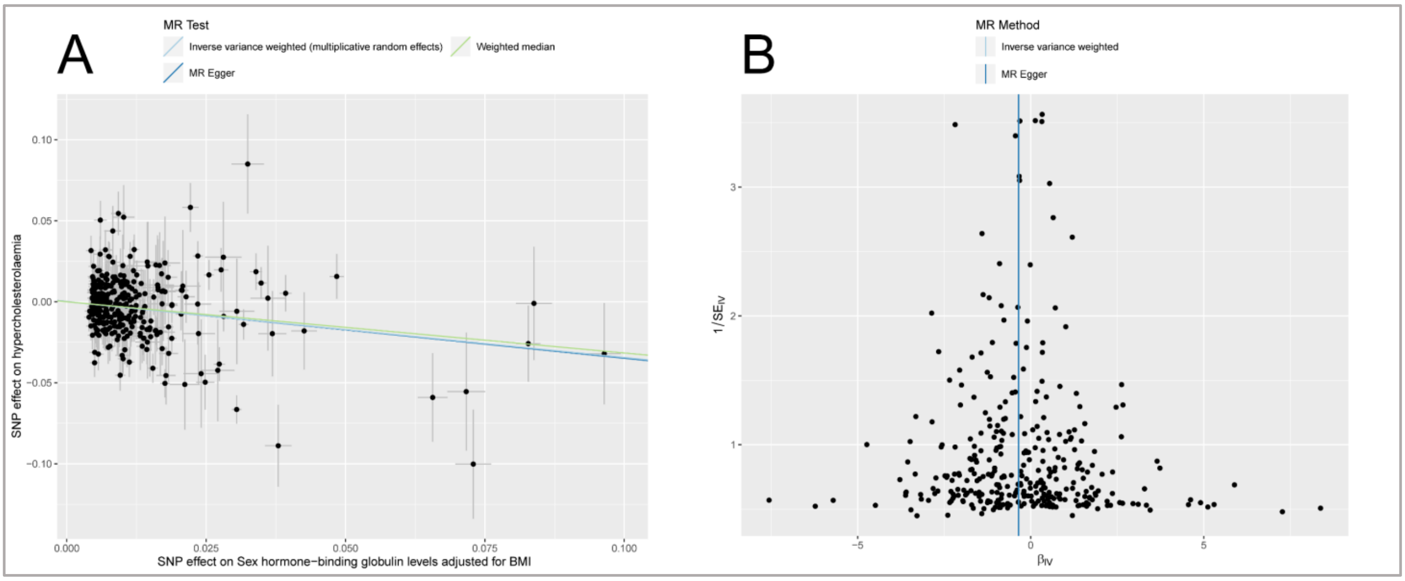

Supplementary Figure S8. Scatter plot and funnel plot for the association of SHBG with HCL. SHBG, sex hormone-binding globulin; HCL, hypercholesterolemia; SNP, single nucleotide polymorphism; SE, standard error; IV, instrumental variable; BMI, body mass index.

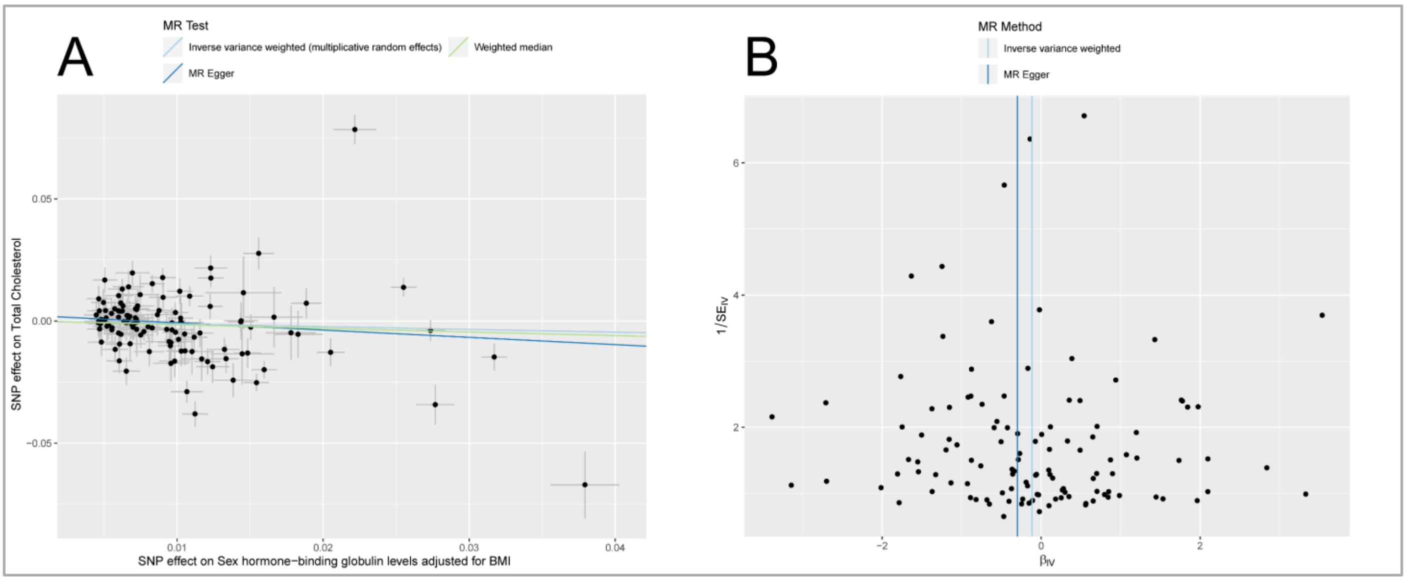

Supplementary Figure S9. Scatter plot and funnel plot for the association of SHBG with TC. SHBG, sex hormone-binding globulin; TC, total cholesterol; SNP, single nucleotide polymorphism; SE, standard error; IV, instrumental variable; BMI, body mass index.

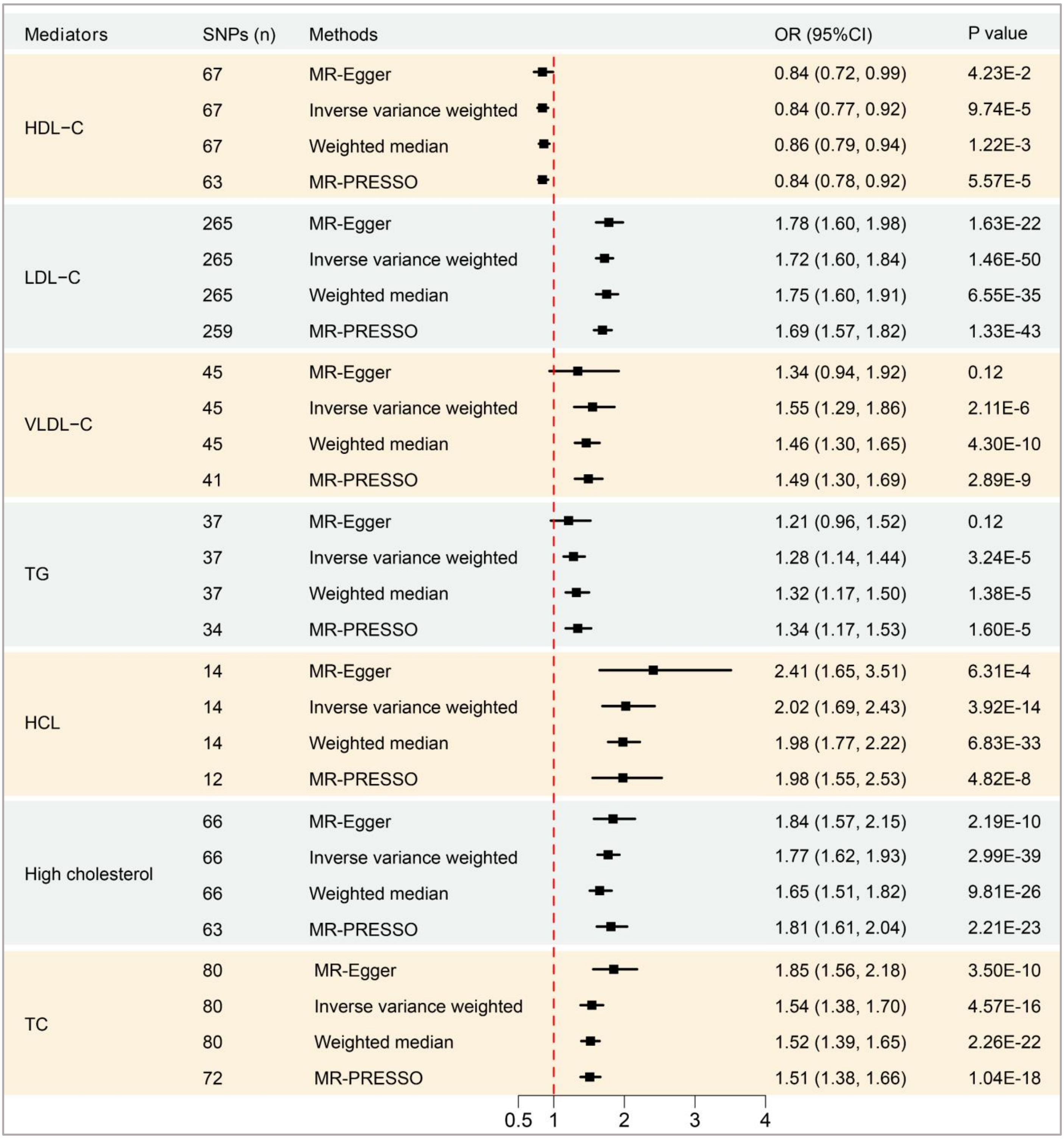

Supplementary Figure S10. Four Mendelian randomization analysis methods used to study the association between mediators and CHD. CHD, coronary heart disease; HDL-C, high-density lipoprotein cholesterol; LDL-C, low-density lipoprotein cholesterol; VLDL-C, very low-density lipoprotein cholesterol; TG, triglycerides; HCL, hypercholesterolemia; TC, total cholesterol; SNPs, single nucleotide polymorphisms; OR, odds ratio; CI, confidence interval.

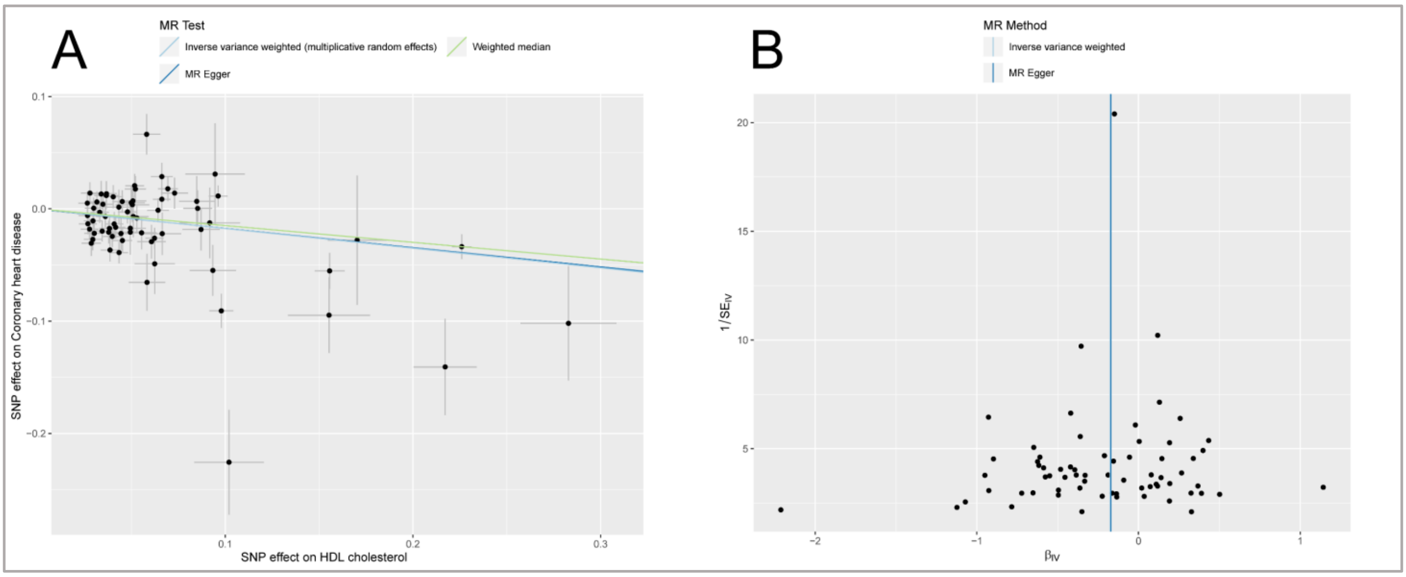

Supplementary Figure S11. Scatter plot and funnel plot for the association of HDL-C with CHD. HDL-C, high-density lipoprotein cholesterol; CHD, coronary heart disease; SNP, single nucleotide polymorphism; SE, standard error; IV, instrumental variable.

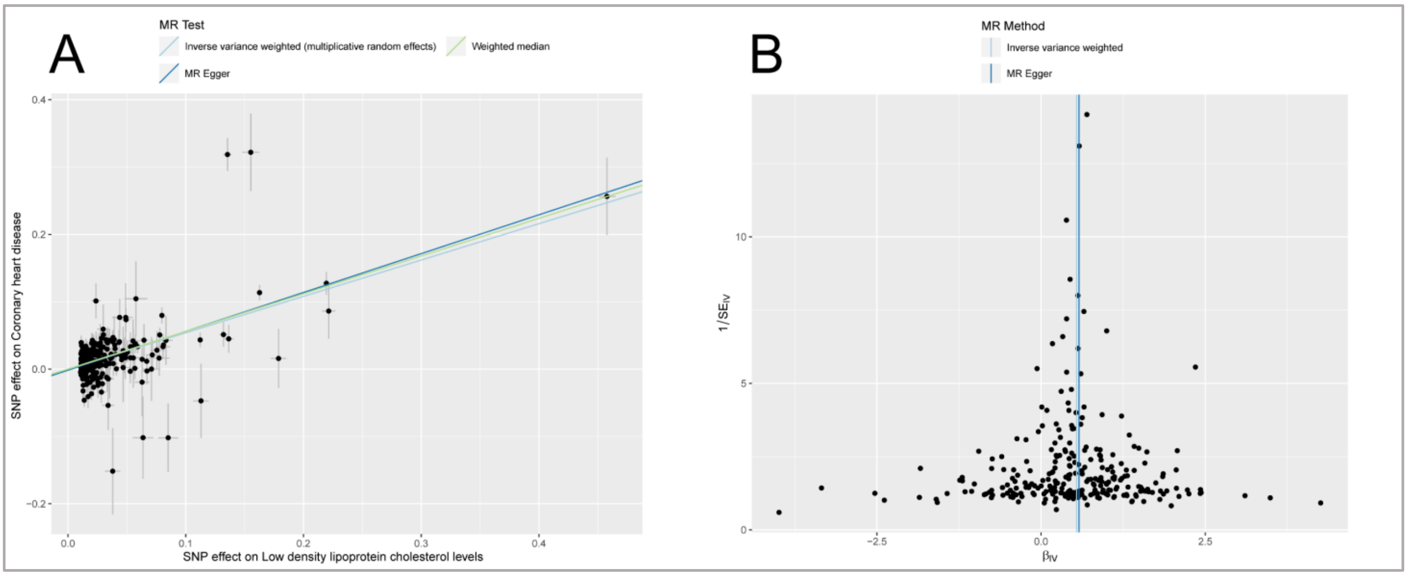

Supplementary Figure S12. Scatter plot and funnel plot for the association of LDL-C with CHD. LDL-C, low-density lipoprotein cholesterol; CHD, coronary heart disease; SNP, single nucleotide polymorphism; SE, standard error; IV, instrumental variable.

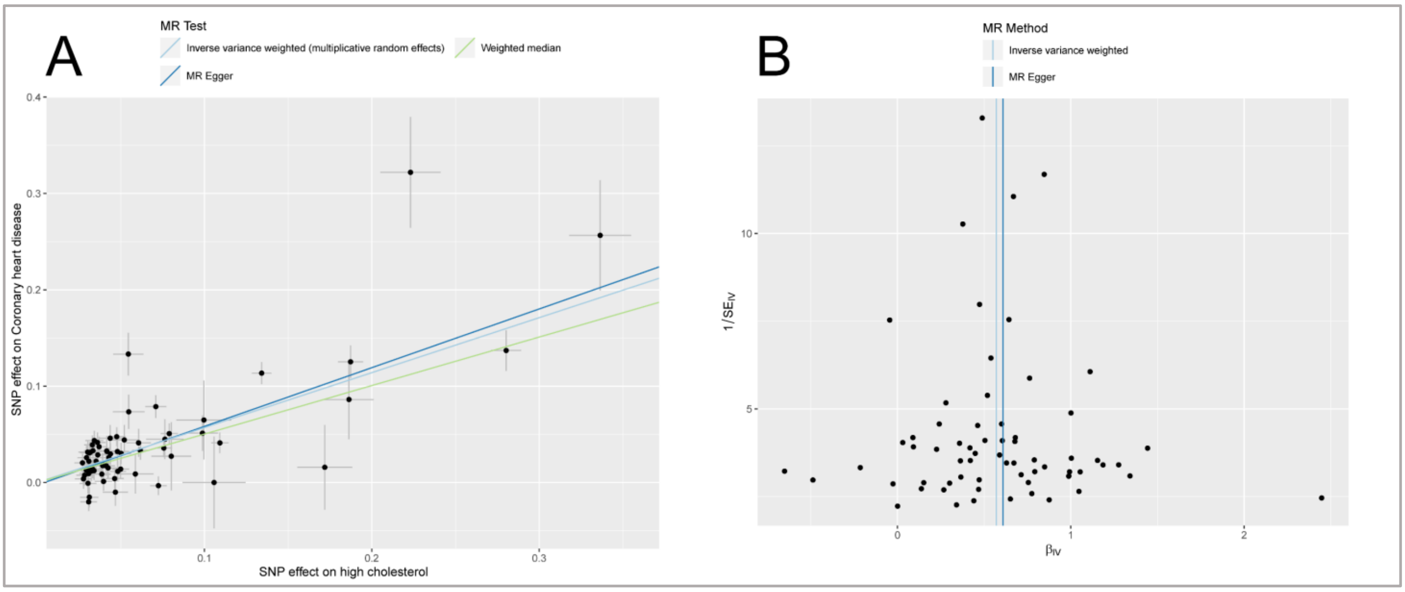

Supplementary Figure S13. Scatter plot and funnel plot for the association of high cholesterol with CHD. CHD, coronary heart disease; SNP, single nucleotide polymorphism; SE, standard error; IV, instrumental variable.

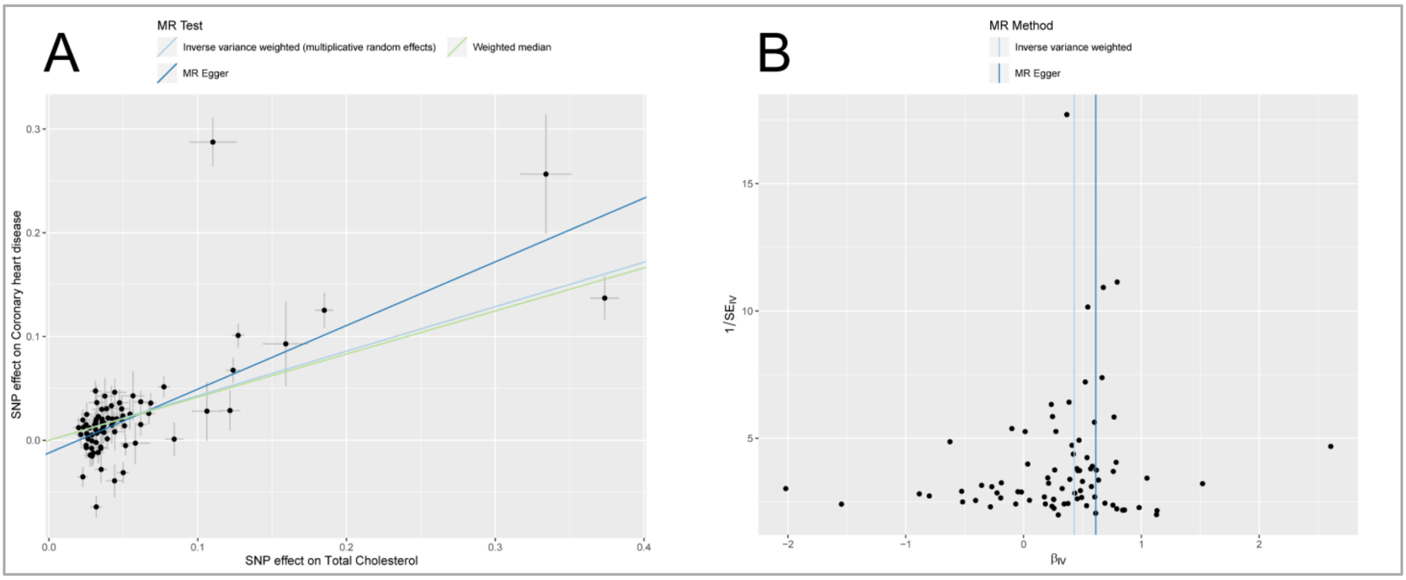

Supplementary Figure S14. Scatter plot and funnel plot for the association of TC with CHD. TC, total cholesterol; CHD, coronary heart disease; SNP, single nucleotide polymorphism; SE, standard error; IV, instrumental variable.

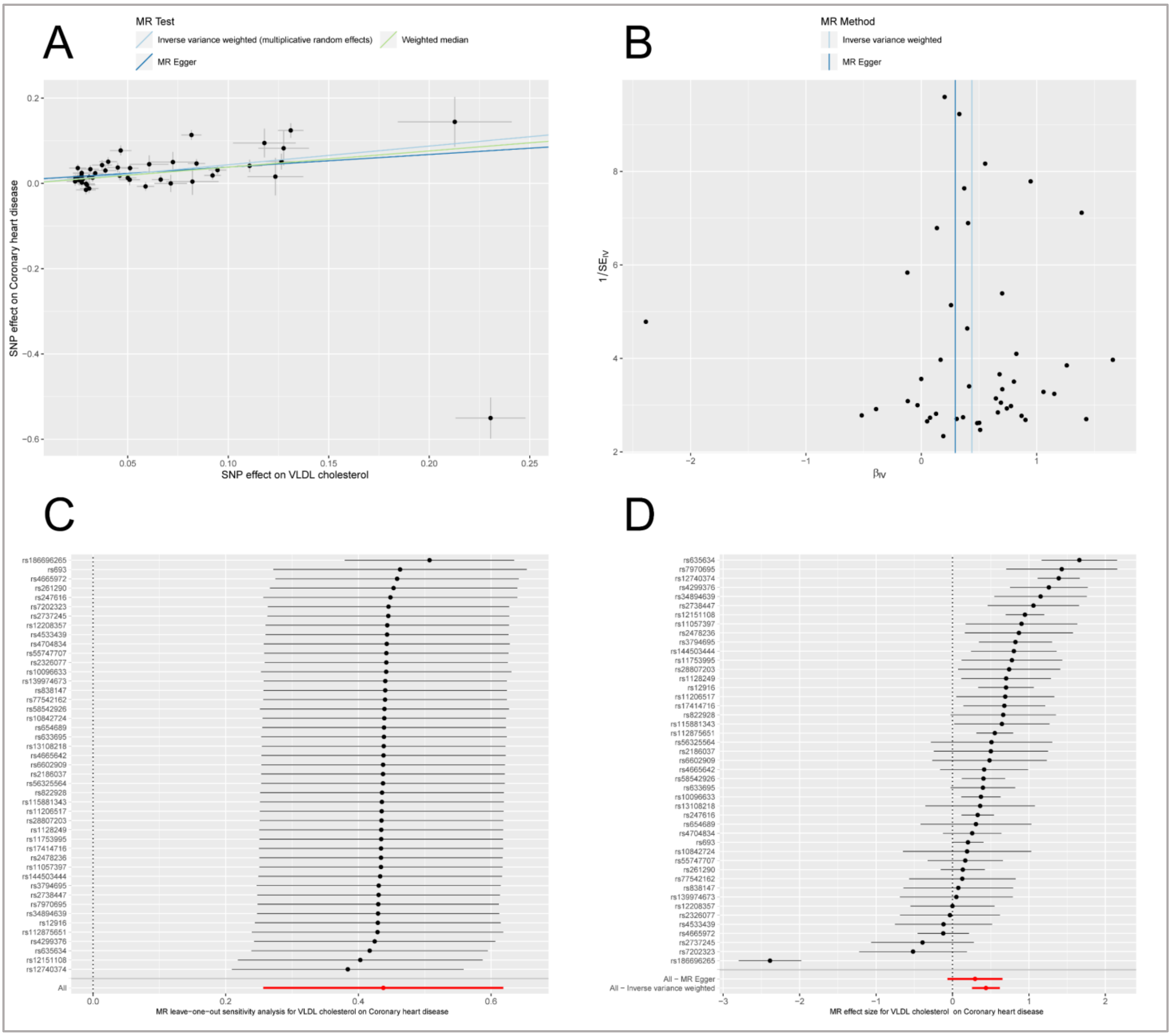

Supplementary Figure S15. Scatter plot, funnel plot, leave-one-out sensitivity analysis and forest plot of a single SNP for the association of VLDL-C with CHD. VLDL-C, very low-density lipoprotein cholesterol; CHD, coronary heart disease; SNP, single nucleotide polymorphism; SE, standard error; IV, instrumental variable.

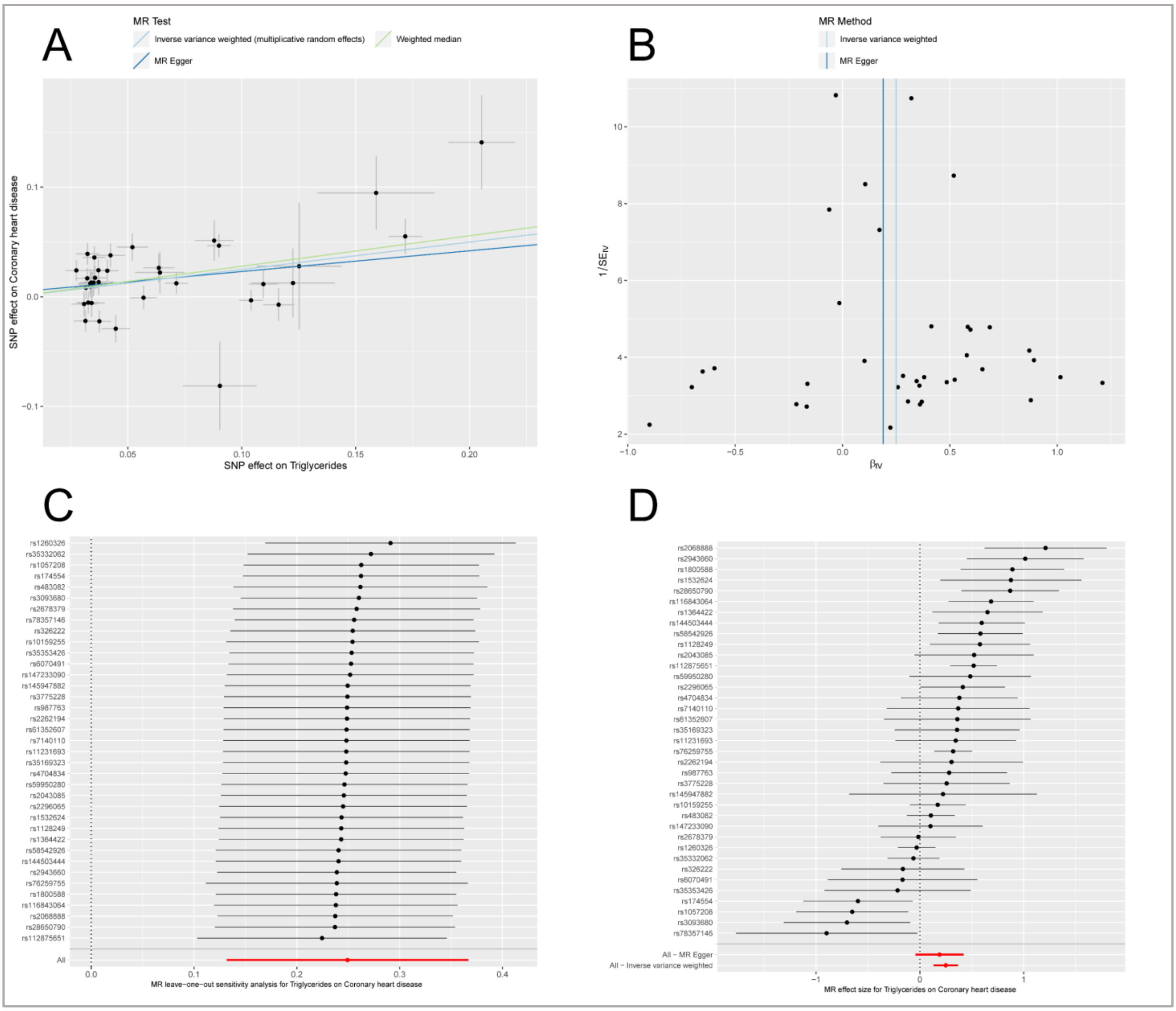

Supplementary Figure S16. Scatter plot, funnel plot, leave-one-out sensitivity analysis and forest plot of a single SNP for the association of TG with CHD. TG, triglycerides; CHD, coronary heart disease; SNP, single nucleotide polymorphism; SE, standard error; IV, instrumental variable.

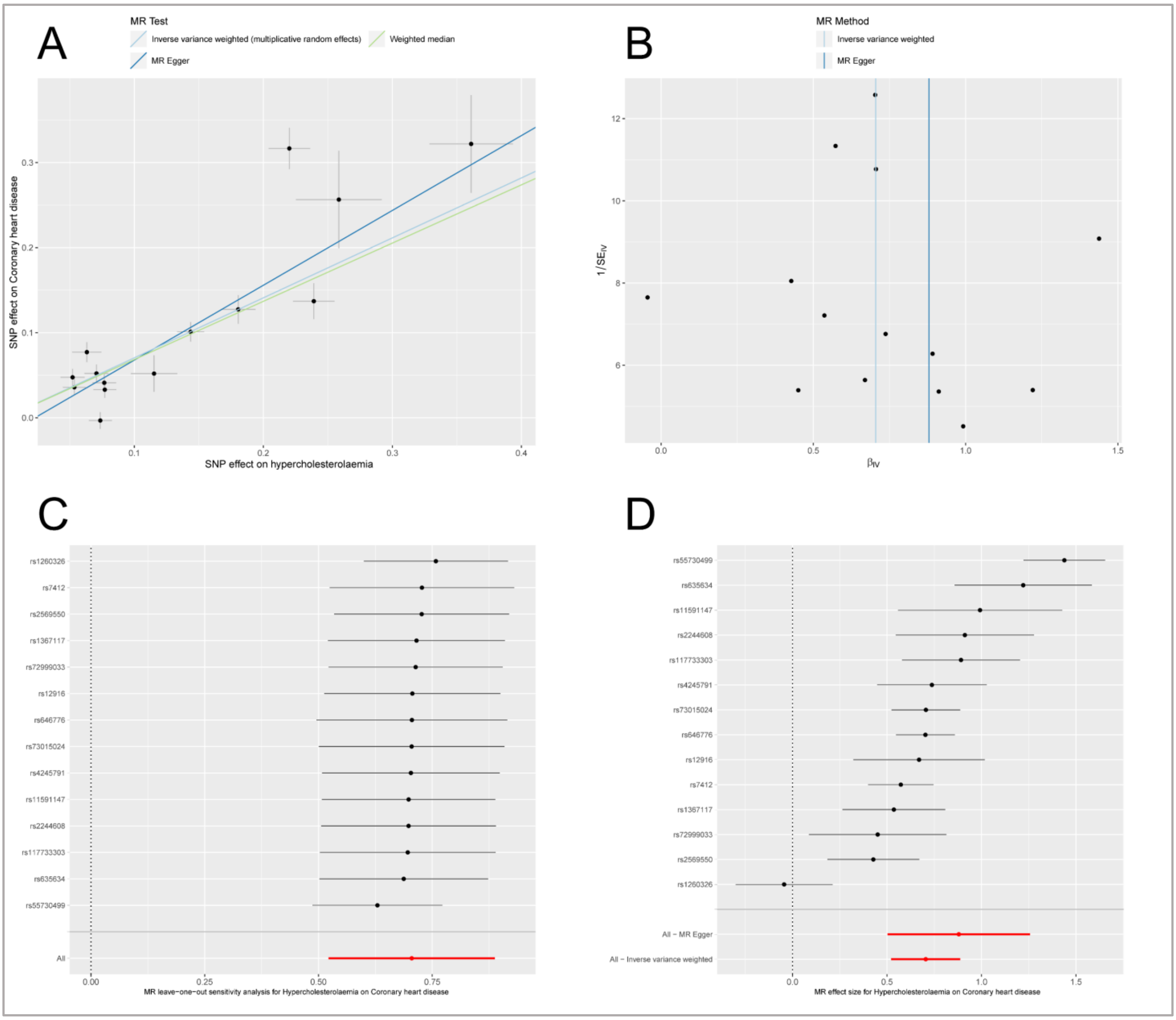

Supplementary Figure S17. Scatter plot, funnel plot, leave-one-out sensitivity analysis and forest plot of a single SNP for the association of HCL with CHD. HCL, hypercholesterolemia; CHD, coronary heart disease; SNP, single nucleotide polymorphism; SE, standard error; IV, instrumental variable.

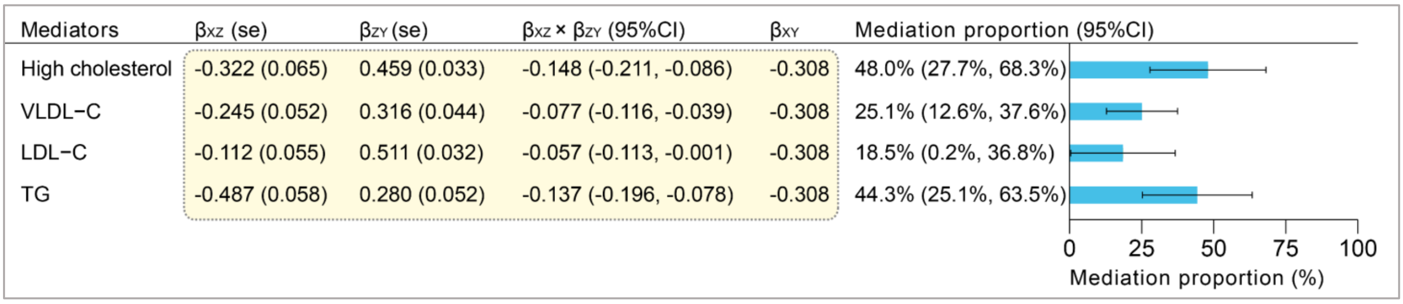

Mediators	β_{XZ} (se)	β_{ZY} (se)	$\beta_{XZ} \times \beta_{ZY}$ (95%CI)	β_{XY}	Mediation proportion (95%CI)
High cholesterol	-0.322 (0.065)	0.459 (0.033)	-0.148 (-0.211, -0.086)	-0.308	48.0% (27.7%, 68.3%)
VLDL−C	-0.245 (0.052)	0.316 (0.044)	-0.077 (-0.116, -0.039)	-0.308	25.1% (12.6%, 37.6%)
LDL−C	-0.112 (0.055)	0.511 (0.032)	-0.057 (-0.113, -0.001)	-0.308	18.5% (0.2%, 36.8%)
TG	-0.487 (0.058)	0.280 (0.052)	-0.137 (-0.196, -0.078)	-0.308	44.3% (25.1%, 63.5%)

Supplementary Figure S18. Mediating effect of blood lipid level in the causality between SHBG and CHD (95% CI for the mediation proportion calculated using the Bootstrap method). SHBG, sex hormone-binding globulin; CHD, coronary heart disease; CI, confidence interval; VLDL-C, very low-density lipoprotein cholesterol; LDL-C, low-density lipoprotein cholesterol; TG, triglycerides; se, standard error.

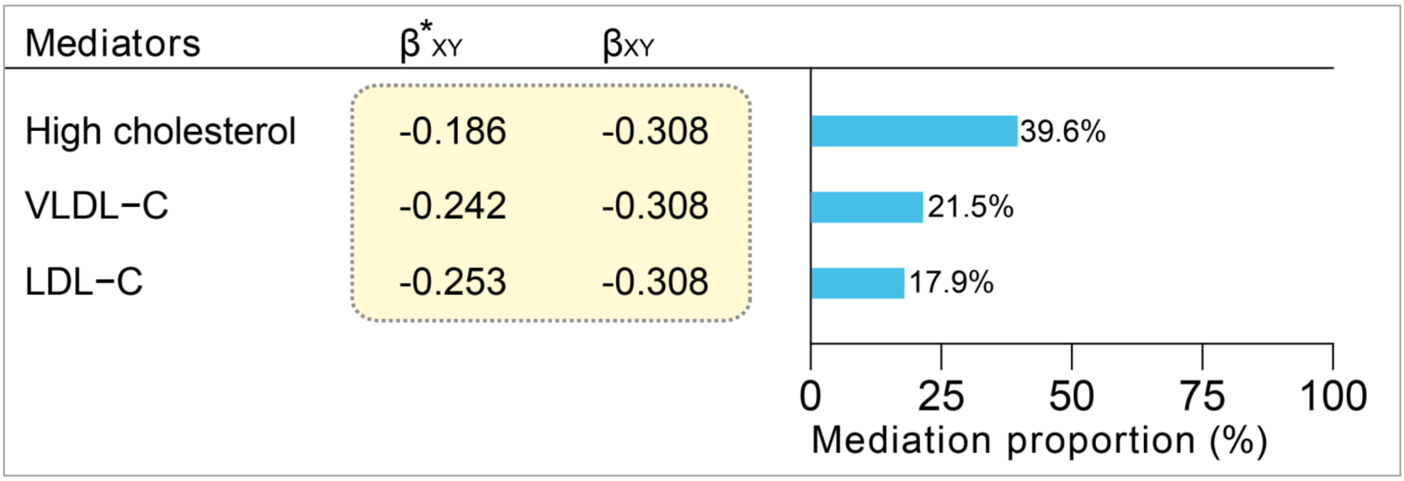

Supplementary Figure S19. Mediating effect of partial mediators that is calculated by difference method in the causality between SHBG and CHD (with GWAS sample overlap between the SHBG and mediators). SHBG, sex hormone-binding globulin; CHD, coronary heart disease; VLDL-C, very low-density lipoprotein cholesterol; LDL-C, low-density lipoprotein cholesterol.

Supplementary Table S1. Detailed information on GWAS summary-level data of the continuous phenotypes

Phenotype	Year of publication	Ethnicity	Consortium	Sample size	Number of SNPs
SHBG	2020	European	UK Biobank	368,929	16,137,123
SHBG (in men)	2020	European	UK Biobank	180,094	16,136,762
SHBG (in women)	2020	European	UK Biobank	188,908	16,140,001
TC	2013	European	GLGC	94,595	2,418,562
TG	2022	European	WFC	78,700	7,892,037
LDL-C	2020	European	UK Biobank	431,167	16,293,344
VLDL-C	2020	European	Nightingale Health	115,078	12,321,875
HDL-C	2022	European	WFC	77,409	7,892,377

GWAS, genome-wide association study; SHBG, sex hormone-binding globulin; TC, total cholesterol; TG, triglycerides; LDL-C, low-density lipoprotein cholesterol; VLDL-C, very low-density lipoprotein cholesterol; HDL-C, high-density lipoprotein cholesterol; GLGC, Global Lipids Genetics Consortium; WFC, Within Family Consortium; SNPs, single nucleotide polymorphisms.

Supplementary Table S2. Detailed information on GWAS summary-level data of the dichotomous phenotypes

Phenotype	Year*	Ethnicity	Consortium	Cases	Controls	Number of SNPs
AF&FL	2023	Finnish	FinnGen consortium	45,766	191,924	20,164,886
Coronary heart disease	2015	77%European	CARDIoGRAMplusC4D	60,801	123,504	9,455,779
Heart failure	2023	Finnish	FinnGen consortium	27,304	349,973	20,170,236
Myocardial infarction	2015	77%European	CARDIoGRAMplusC4D	43,676	128,199	9,289,492
Hypertension	2023	Finnish	FinnGen consortium	111,581	265,626	20,170,234
High cholesterol	2018	European	MRC-IEU	56,753	406,180	9,851,867
Hypercholesterolemia	2018	European	MRC-IEU	22,622	440,388	9,851,867

GWAS, genome-wide association study; AF&FL, atrial fibrillation & flutter; MRC-IEU, Medical Research Council Integrative Epidemiology Unit (University of Bristol); SNPs, single nucleotide polymorphisms.

*Year of publication.

Supplementary Table S3. Characteristics of GWAS summary-level data for continuous phenotypes

Phenotype	Units*	Description of phenotype	Details of assay†
SHBG	SD (nmol/L)	Serum SHBG levels Adjusted for BMI	From blood sample collected at initial visit. Measured by two step competitive analysis on a Beckman Coulter Unicel Dxl 800.
TC	SD (mg/dL)	Serum total cholesterol levels	This GWAS array includes 23 studies involving participants of European ancestry, with variations in the detection methods across different studies.
TG	SD	Serum triglyceride levels	Participants were not subjected to treatment for blood triglyceride levels. This GWAS involves 17 cohorts, with variations in the detection methods across different cohorts.
LDL-C	SD (mmol/L)	Serum low-density lipoprotein cholesterol levels	Blood samples from non-fasting participants of the UK Biobank. Assessed by enzymatic protective selection analysis on a Beckman Coulter AU5800.
VLDL-C	SD (mmol/L)	Plasma very low-density lipoprotein cholesterol levels	118,461 baseline plasma samples from the UK Biobank. The plasma samples were measured using six 500MHz NMR spectrometers (Bruker AVANCE IIIHD).
HDL-C	SD	Serum high-density lipoprotein cholesterol levels	Participants were not subjected to treatment for blood HDL-C levels. This GWAS involves 17 cohorts, with variations in the detection methods across different cohorts.

GWAS, genome-wide association study; SHBG, sex hormone-binding globulin; TC, total cholesterol; TG, triglycerides; LDL-C, low-density lipoprotein cholesterol; VLDL-C, very low-density lipoprotein cholesterol; HDL-C, high-density lipoprotein cholesterol; SD, standard deviation; BMI, body mass index; NMR, nuclear magnetic resonance.

*The effect size is reported in standard deviation (SD) change, units in parentheses represents the original measurement of the biomarker level.

†VLDL-C is measured using NMR technology, not chemical methods.

Supplementary Table S4. Diagnosis and definition of dichotomous phenotypes (diseases)

Phenotype (disease)	Diagnosis and definition
AF&FL	ICD-8: 42792<br>ICD-9: 4273<br>ICD-10: I48-Atrial fibrillation and flutter
Heart failure	ICD-8: 42700, 42710, 428, 7824<br>ICD-9: 4029B, 428<br>ICD-10: I11-Hypertensive heart disease, I13.0-Hypertensive heart and renal disease with (congestive) heart failure, I13.2-Hypertensive heart and renal disease with both (congestive) heart failure and renal failure, I50-Heart failure
Hypertension	ICD-8: 400, 401, 402, 403, 404<br>ICD-9: 4019X, 4029A, 4029B, 4039A, 4040A, 4059A, 4059B, 4372A, 4059X<br>ICD-10: I10-Essential hypertension, I11-Hypertensive heart disease, I12-Hypertensive renal disease, I13-Hypertensive heart and renal disease, I15-Secondary hypertension, I67.4-Hypertension encephalopathy
Hypercholesterolemia	ICD-10: E78.0-Pure hypercholesterolemia
High cholesterol	Self-reported: high cholesterol
Coronary heart disease	Diagnosis of coronary heart disease (for example, myocardial infarction, acute coronary syndrome, chronic stable angina or coronary stenosis of >50%)
Myocardial infarction	\

AF&FL, atrial fibrillation & flutter; ICD, International Classification of Diseases.

Supplementary Table S5. Instrumental variables used for the Mendelian randomization analysis of the effect of serum SHBG levels on CHD risk. SHBG, sex hormone-binding globulin; CHD, coronary heart disease; SNP, single nucleotide polymorphism; CHR, chromosome; POS, position; EA, effect allele; OA, other allele; Beta, effect size estimate; Se, standard error. See separate Excel file. **See separate Excel file worksheet ST 5.**

Supplementary Table S6. Instrumental variables used for the Mendelian randomization analysis of the effect of serum SHBG levels on high cholesterol risk. SHBG, sex hormone-binding globulin; SNP, single nucleotide polymorphism; CHR, chromosome; POS, position; EA, effect allele; OA, other allele; Beta, effect size estimate; Se, standard error. See separate Excel file. **See separate Excel file worksheet ST 6.**

Supplementary Table S7. Instrumental variables used for the Mendelian randomization analysis of the effect of serum SHBG levels on hypercholesterolemia risk. SHBG, sex hormone-binding globulin; SNP, single nucleotide polymorphism; CHR, chromosome; POS, position; EA, effect allele; OA, other allele; Beta, effect size estimate; Se, standard error. See separate Excel file. **See separate Excel file worksheet ST 7.**

Supplementary Table S8. Instrumental variables used for the Mendelian randomization analysis of the effect of serum SHBG levels on VLDL-C levels. SHBG, sex hormone-binding globulin; VLDL-C, very low-density lipoprotein cholesterol; SNP, single nucleotide polymorphism; CHR, chromosome; POS, position; EA, effect allele; OA, other allele; Beta, effect size estimate; Se, standard error. See separate Excel file. **See separate Excel file worksheet ST 8.**

Supplementary Table S9. Instrumental variables used for the Mendelian randomization analysis of the effect of serum SHBG levels on LDL-C levels. SHBG, sex hormone-binding globulin; LDL-C, low-density lipoprotein cholesterol; SNP, single nucleotide polymorphism; CHR, chromosome; POS, position; EA, effect allele; OA, other allele; Beta, effect size estimate; Se, standard error. See separate Excel file. **See separate Excel file worksheet ST 9.**

Supplementary Table S10. Instrumental variables used for the Mendelian randomization analysis of the effect of serum SHBG levels on triglycerides levels. SHBG, sex hormone-binding globulin; SNP, single nucleotide polymorphism; CHR, chromosome; POS, position; EA, effect allele; OA, other allele; Beta, effect size estimate; Se, standard error. See separate Excel file. **See separate Excel file worksheet ST 10.**

Supplementary Table S11. Instrumental variables used for the Mendelian randomization analysis of the effect of serum SHBG levels on HDL-C levels. SHBG, sex hormone-binding globulin; HDL-C, high-density lipoprotein cholesterol; SNP, single nucleotide polymorphism; CHR, chromosome; POS, position; EA, effect allele; OA, other allele; Beta, effect size estimate; Se, standard error. See separate Excel file. **See separate Excel file worksheet ST 11.**

Supplementary Table S12. Instrumental variables used for the Mendelian randomization analysis of the effect of serum SHBG levels on total cholesterol levels. SHBG, sex hormone-binding globulin; SNP, single nucleotide polymorphism; CHR, chromosome; POS, position; EA, effect allele; OA, other allele; Beta, effect size estimate; Se, standard error. See separate Excel file. **See separate Excel file worksheet ST 12.**

Supplementary Table S13. Instrumental variables used for the Mendelian randomization analysis of the effect of high cholesterol on CHD risk. CHD, coronary heart disease; SNP, single nucleotide polymorphism; CHR, chromosome; POS, position; EA, effect allele; OA, other allele; Beta, effect size estimate; Se, standard error. See separate Excel file. **See separate Excel file worksheet ST 13.**

Supplementary Table S14. Instrumental variables used for the Mendelian randomization analysis of the effect of hypercholesterolemia on CHD risk. CHD, coronary heart disease; SNP, single nucleotide polymorphism; CHR, chromosome; POS, position; EA, effect allele; OA, other allele; Beta, effect size estimate; Se, standard error. See separate Excel file. **See separate Excel file worksheet ST 14.**

Supplementary Table S15. Instrumental variables used for the Mendelian randomization analysis of the effect of VLDL-C levels on CHD risk. VLDL-C, very low-density lipoprotein cholesterol; CHD, coronary heart disease; SNP, single nucleotide polymorphism; CHR, chromosome; POS, position; EA, effect allele; OA, other allele; Beta, effect size estimate; Se, standard error. See separate Excel file. **See separate Excel file worksheet ST 15.**

Supplementary Table S16. Instrumental variables used for the Mendelian randomization analysis of the effect of LDL-C levels on CHD risk. LDL-C, low-density lipoprotein cholesterol; CHD, coronary heart disease; SNP, single nucleotide polymorphism; CHR, chromosome; POS, position; EA, effect allele; OA, other allele; Beta, effect size estimate; Se, standard error. See separate Excel file. **See separate Excel file worksheet ST 16.**

Supplementary Table S17. Instrumental variables used for the Mendelian randomization analysis of the effect of triglycerides levels on CHD risk. CHD, coronary heart disease; SNP, single nucleotide polymorphism; CHR, chromosome; POS, position; EA, effect allele; OA, other allele; Beta, effect size estimate; Se, standard error. See separate Excel file. **See separate Excel file worksheet ST 17.**

Supplementary Table S18. Instrumental variables used for the Mendelian randomization analysis of the effect of HDL-C levels on CHD risk. HDL-C, high-density lipoprotein cholesterol; CHD, coronary heart disease; SNP, single nucleotide polymorphism; CHR, chromosome; POS, position; EA, effect allele; OA, other allele; Beta, effect size estimate; Se, standard error. See separate Excel file. **See separate Excel file worksheet ST 18.**

Supplementary Table S19. Instrumental variables used for the Mendelian randomization analysis of the effect of total cholesterol levels on CHD risk. CHD, coronary heart disease; SNP, single nucleotide polymorphism; CHR, chromosome; POS, position; EA, effect allele; OA, other allele; Beta, effect size estimate; Se, standard error. See separate Excel file. **See separate Excel file worksheet ST 19.**

Supplementary Table S20. Instrumental variables used for the multivariable Mendelian randomization analysis of the effect of serum SHBG levels and high cholesterol on CHD risk. SHBG, sex hormone-binding globulin; CHD, coronary heart disease; SNP, single nucleotide polymorphism; CHR, chromosome; POS, position; EA, effect allele; OA, other allele; Beta, effect size estimate; Se, standard error. See separate Excel file. **See separate Excel file worksheet ST 20.**

Supplementary Table S21. Instrumental variables used for the multivariable Mendelian randomization analysis of the effect of serum SHBG levels and hypercholesterolemia on CHD risk. SHBG, sex hormone-binding globulin; CHD, coronary heart disease; SNP, single nucleotide polymorphism; CHR, chromosome; POS, position; EA, effect allele; OA, other allele; Beta, effect size estimate; Se, standard error. See separate Excel file. **See separate Excel file worksheet ST 21.**

Supplementary Table S22. Instrumental variables used for the multivariable Mendelian randomization analysis of the effect of serum SHBG and VLDL-C levels on CHD risk. SHBG, sex hormone-binding globulin; VLDL-C, very low-density lipoprotein cholesterol; CHD, coronary heart disease; SNP, single nucleotide polymorphism; CHR, chromosome; POS, position; EA, effect allele; OA, other allele; Beta, effect size estimate; Se, standard error. See separate Excel file. **See separate Excel file worksheet ST 22.**

Supplementary Table S23. Instrumental variables used for the multivariable Mendelian randomization analysis of the effect of serum SHBG and LDL-C levels on CHD risk. SHBG, sex hormone-binding globulin; LDL-C, low-density lipoprotein cholesterol; CHD, coronary heart disease; SNP, single nucleotide polymorphism; CHR, chromosome; POS, position; EA, effect allele; OA, other allele; Beta, effect size estimate; Se, standard error. See separate Excel file. **See separate Excel file worksheet ST 23.**

Supplementary Table S24. Instrumental variables used for the multivariable Mendelian randomization analysis of the effect of serum SHBG and triglycerides levels on CHD risk. SHBG, sex hormone-binding globulin; CHD, coronary heart disease; SNP, single nucleotide polymorphism; CHR, chromosome; POS, position; EA, effect allele; OA, other allele; Beta, effect size estimate; Se, standard error. See separate Excel file. **See separate Excel file worksheet ST 24.**

Supplementary Table S25. Instrumental variables used for the multivariable Mendelian randomization analysis of the effect of serum SHBG and HDL-C levels on CHD risk. SHBG, sex hormone-binding globulin; HDL-C, high-density lipoprotein cholesterol; CHD, coronary heart disease; SNP, single nucleotide polymorphism; CHR, chromosome; POS, position; EA, effect allele; OA, other allele; Beta, effect size estimate; Se, standard error. See separate Excel file. **See separate Excel file worksheet ST 25.**

Supplementary Table S26. Two sensitivity analyses for the causality between SHBG and five CVDs

Outcome	Cochran's Q test		MR-Egger intercept test	
	Q statistic	P-value	Intercept	P-value
AF&FL	351.00	5.96E-36	-7.23E-4	0.66
Heart failure	533.29	1.11E-9	1.11E-3	0.40
Hypertension	1480.65	2.41E-138	-2.09E-3	0.12
Coronary heart disease	713.07	5.71E-28	-2.19E-3	0.17
Myocardial infarction	608.54	4.25E-17	-1.74E-3	0.28

SHBG, sex hormone-binding globulin; CVDs, cardiovascular diseases; AF&FL, atrial fibrillation & flutter.

Supplementary Table S27. Four Mendelian randomization analysis methods used to study the reverse causality between SHBG, mediators and CHD pairwise

Exposure	Outcome	Method	β (95% CI)	OR (95% CI)	P-value
CHD	SHBG	IVW	-0.019 (-0.031, -0.007)	/	2.63E-3
		Weighted Median	-0.012 (-0.020, -0.005)	/	9.71E-4
		MR-Egger	-0.015 (-0.044, 0.014)	/	0.317
		MR-PRESSO	-0.017 (-0.026, -0.007)	/	1.03E-3
CHD	HDL-C	IVW	-0.048 (-0.106, 0.011)	/	0.111
		Weighted Median	-0.005 (-0.044, 0.034)	/	0.802
		MR-Egger	0.022 (-0.115, 0.159)	/	0.758
		MR-PRESSO	-0.034 (-0.068, 0.001)	/	5.48E-2
CHD	LDL-C	IVW	0.259 (0.073, 0.444)	/	6.34E-3
		Weighted Median	-0.026 (-0.042, -0.011)	/	5.69E-4
		MR-Egger	0.370 (-0.063, 0.804)	/	0.103
		MR-PRESSO	0.088 (0.046, 0.130)	/	4.82E-5
CHD	VLDL-C	IVW	0.022 (-0.093, 0.137)	/	0.710
		Weighted Median	-0.054 (-0.085, -0.023)	/	6.51E-4
		MR-Egger	-0.140 (-0.403, 0.124)	/	0.305
		MR-PRESSO	-0.035 (-0.068, -0.001)	/	4.15E-2
CHD	TG	IVW	0.001 (-0.048, 0.051)	/	0.959
		Weighted Median	-0.044 (-0.077, -0.010)	/	1.08E-2
		MR-Egger	-0.059 (-0.173, 0.055)	/	0.320
		MR-PRESSO	-0.010 (-0.042, 0.021)	/	0.525
CHD	high cholesterol	IVW	0.317 (0.166, 0.468)	1.37 (1.18, 1.60)	3.79E-5
		Weighted Median	0.096 (0.055, 0.138)	1.10 (1.06, 1.15)	6.11E-6
		MR-Egger	0.394 (0.044, 0.745)	1.48 (1.04, 2.11)	3.44E-2
		MR-PRESSO	0.181 (0.110, 0.253)	1.20 (1.12, 1.29)	6.73E-7
CHD	HCL	IVW	0.501 (0.375, 0.627)	1.65 (1.45, 1.87)	7.06E-15
		Weighted Median	0.344 (0.267, 0.422)	1.41 (1.31, 1.52)	2.51E-18
		MR-Egger	0.651 (0.362, 0.939)	1.92 (1.44, 2.56)	9.63E-5
		MR-PRESSO	0.358 (0.245, 0.471)	1.43 (1.28, 1.60)	5.31E-10
CHD	TC	IVW	0.168 (-0.056, 0.392)	/	0.141
		Weighted Median	-0.035 (-0.066, -0.004)	/	2.80E-2
		MR-Egger	-0.029 (-0.608, 0.551)	/	0.924
		MR-PRESSO	0.044 (0.0001, 0.087)	/	4.94E-2
HDL-C	SHBG	IVW	0.033 (0.005, 0.061)	/	2.27E-2

		Weighted Median	-0.008 (-0.015, -0.0001)	/	4.60E-2
		MR-Egger	-0.016 (-0.065, 0.032)	/	0.508
		MR-PRESSO	0.036 (0.021, 0.050)	/	1.24E-6
LDL-C	SHBG	IVW	-0.024 (-0.048, 0.001)	/	6.21E-2
		Weighted Median	-0.021 (-0.027, -0.015)	/	3.39E-11
		MR-Egger	-0.019 (-0.051, 0.013)	/	0.248
		MR-PRESSO	-0.014 (-0.020, -0.008)	/	2.06E-5
VLDL-C	SHBG	IVW	-0.053 (-0.102, -0.003)	/	3.72E-2
		Weighted Median	-0.020 (-0.033, -0.007)	/	2.58E-3
		MR-Egger	0.010 (-0.078, 0.097)	/	0.831
		MR-PRESSO	-0.038 (-0.050, -0.025)	/	7.21E-9
TG	SHBG	IVW	-0.089 (-0.150, -0.028)	/	4.26E-3
		Weighted Median	-0.023 (-0.035, -0.012)	/	8.26E-5
		MR-Egger	-0.052 (-0.171, 0.067)	/	0.397
		MR-PRESSO	-0.069 (-0.095, -0.042)	/	5.73E-7
high cholesterol	SHBG	IVW	-0.048 (-0.089, -0.006)	/	2.38E-2
		Weighted Median	-0.010 (-0.020, -0.0005)	/	3.98E-2
		MR-Egger	-0.012 (-0.083, 0.060)	/	0.748
		MR-PRESSO	-0.034 (-0.048, -0.020)	/	1.41E-6
HCL	SHBG	IVW	-0.049 (-0.109, 0.010)	/	0.106
		Weighted Median	-0.020 (-0.031, -0.010)	/	2.06E-4
		MR-Egger	-0.002 (-0.121, 0.117)	/	0.976
		MR-PRESSO	-0.044 (-0.074, -0.014)	/	3.61E-3
TC	SHBG	IVW	-0.017 (-0.047, 0.014)	/	0.287
		Weighted Median	-0.025 (-0.034, -0.016)	/	1.08E-7
		MR-Egger	-0.011 (-0.059, 0.038)	/	0.663
		MR-PRESSO	-0.017 (-0.027, -0.007)	/	1.10E-3

SHBG, sex hormone-binding globulin; CHD, coronary heart disease; HDL-C, high-density lipoprotein cholesterol; LDL-C, low-density lipoprotein cholesterol; VLDL-C, very low-density lipoprotein cholesterol; TG, triglycerides; HCL, hypercholesterolemia; TC, total cholesterol; IVW, inverse variance weighted; CI, confidence interval; OR, odds ratio.

Supplementary Table S28. Two sensitivity analyses for the reverse causality between SHBG, mediators and CHD pairwise

Exposure	Outcome	Cochran's Q test		MR-Egger intercept test	
		Q statistic	P-value	Intercept	P-value
CHD	SHBG	479.38	2.54E-78	-4.36E-4	0.77
CHD	HDL-C	285.03	1.99E-41	-7.47E-3	0.28
CHD	LDL-C	20606.50	<1.00E-300	-1.21E-2	0.58
CHD	VLDL-C	1696.88	<1.00E-300	1.76E-2	0.19
CHD	TG	186.74	5.40E-23	6.51E-3	0.26
CHD	high cholesterol	1946.27	<1.00E-300	-8.39E-3	0.64
CHD	HCL	423.22	3.74E-68	-1.63E-2	0.27
CHD	TC	2345.80	<1.00E-300	2.21E-2	0.48
HDL-C	SHBD	3139.31	<1.00E-300	3.66E-3	0.02
LDL-C	SHBD	21278.26	<1.00E-300	-2.86E-4	0.66
VLDL-C	SHBD	5693.97	<1.00E-300	-4.22E-3	0.10
TG	SHBD	4555.26	<1.00E-300	-2.56E-3	0.48
High cholesterol	SHBD	7764.21	<1.00E-300	-2.59E-3	0.24
HCL	SHBD	2037.05	<1.00E-300	-6.15E-3	0.38
TC	SHBD	4936.07	<1.00E-300	-4.27E-4	0.76

SHBG, sex hormone-binding globulin; CHD, coronary heart disease; HDL-C, high-density lipoprotein cholesterol; LDL-C, low-density lipoprotein cholesterol; VLDL-C, very low-density lipoprotein cholesterol; TG, triglycerides; HCL, hypercholesterolemia; TC, total cholesterol.

Supplementary Table S29. Effect estimates of the causality between SHBG and four mediators (corrected for bias primarily induced by sample overlap using MRlap)

Mediators	SNPs	Observed β (95%CI)	Observed p	Corrected β (95%CI)	Corrected p	Test difference	P_difference
LDL-C	758	-0.0534 (-0.0801, -0.0267)	9.02E-5	-0.0534 (-0.0803, -0.0265)	9.81E-5	-0.045	0.964
VLDL-C	759	-0.1023 (-0.1256, -0.0790)	9.04E-18	-0.1023 (-0.1258, -0.0788)	1.20E-17	-0.012	0.991
High cholesterol	749	-0.0484 (-0.0621, -0.0347)	4.94E-12	-0.0484 (-0.0621, -0.0347)	3.60E-12	-0.006	0.995
HCL	737	-0.0255 (-0.0347, -0.0163)	4.57E-8	-0.0255 (-0.0347, -0.0163)	5.29E-8	-0.087	0.931

SHBG, sex hormone-binding globulin; LDL-C, low-density lipoprotein cholesterol; VLDL-C, very low-density lipoprotein cholesterol; HCL, hypercholesterolemia; SNPs, single nucleotide polymorphisms; CI, confidence interval.

Supplementary Table S30. Two sensitivity analyses for the causality between mediators and CHD

Mediators	Cochran's Q test		MR-Egger intercept test	
	Q statistic	P-value	Intercept	P-value
HDL-C	219.45	2.31E-18	-2.67E-4	0.96
LDL-C	681.41	1.14E-38	-1.63E-3	0.35
VLDL-C	356.27	1.77E-50	8.77E-3	0.37
TG	116.97	1.71E-10	4.06E-3	0.56
HCL	99.92	1.72E-15	-2.02E-2	0.32
High cholesterol	186.97	9.72E-14	4.55E-3	0.57
TC	369.64	3.90E-39	-1.22E-2	8.72E-3

CHD, coronary heart disease; HDL-C, high-density lipoprotein cholesterol; LDL-C, low-density lipoprotein cholesterol; VLDL-C, very low-density lipoprotein cholesterol; TG, triglycerides; HCL, hypercholesterolemia; TC, total cholesterol.

Supplementary Table S31. F-statistics calculation and Cochran's Q test for MVMR analyses

Exposure (Variable 1)	Outcome	Mediator (Variable 2)	F-statistics for instrument strength		P-value of Q-Statistic
			Variable 1	Variable 2	
SHBG	CHD	HDL-C	102.0	16.9	8.25E-24
		LDL-C	106.1	140.6	3.03E-35
		VLDL-C	91.8	13.7	2.88E-26
		TG	58.0	10.4	1.78E-22
		High cholesterol	106.7	17.5	7.44E-12
		HCL	38.2	3.8	2.76E-5

MVMR, multivariable Mendelian randomization; SHBG, sex hormone-binding globulin; CHD, coronary heart disease; HDL-C, high-density lipoprotein cholesterol; LDL-C, low-density lipoprotein cholesterol; VLDL-C, very low-density lipoprotein cholesterol; TG, triglycerides; HCL, hypercholesterolemia.

Supplementary Table S32. Two sensitivity analyses for sex-specific causality between SHBG and CHD

Exposure	Cochran's Q test		MR-Egger intercept test	
	Q statistic	P-value	Intercept	P-value
SHBG (in men)	443.02	1.26E-20	-4.53E-3	4.84E-2
SHBG (in women)	533.18	7.49E-25	-4.42E-3	4.53E-2

SHBG, sex hormone-binding globulin; CHD, coronary heart disease.

Supplementary Table S33. STROBE-MR checklist of recommended items to address in reports of Mendelian randomization study

Item No.	Section	Checklist item	
**Title and Abstract**			
1	Title and Abstract	Indicate mendelian randomization (MR) as the study's design in the title and/or the abstract if that is a main purpose of the study.	**Page 1-2**
**Introduction**			
2	Background	Explain the scientific background and rationale for the reported study. What is the exposure? Is a potential causal relationship between exposure and outcome plausible? Justify why MR is a helpful method to address the study question.	**Page 3**
3	Objectives	State specific objectives clearly, including prespecified causal hypotheses (if any). State that MR is a method that, under specific assumptions, intends to estimate causal effects.	**Page 4**
**Methods**			
4	Study design and data sources	Present key elements of the study design early in the article. Consider including a table listing sources of data for all phases of the study. For each data source contributing to the analysis, describe the following:	
	a	Setting: Describe the study design and the underlying population, if possible. Describe the setting, locations, and relevant dates, including periods of recruitment, exposure, follow-up, and data collection, when available.	**Page 4, 5**
	b	Participants: Report the eligibility criteria and the sources and methods of selection of participants. Report the sample size and whether any power or sample size calculations were carried out prior to the main analysis.	**Page 4, 5**<br>**Table S1, S2**
	c	Describe measurement, quality control, and selection of genetic variants.	**Page 6**

	d	For each exposure, outcome, and other relevant variables, describe methods of assessment and diagnostic criteria for diseases.	**Table S1-S4**<br>**Text S1**
	e	Provide details of ethics committee approval and participant informed consent, if relevant.	**Page 4**
5	Assumptions	Explicitly state the 3 core instrumental variable (IV) assumptions for the main analysis (relevance, independence, and exclusion restriction), as well assumptions for any additional or sensitivity analysis.	**Page 3**
6	Statistical methods: main analysis	Describe statistical methods and statistics used.	
	a	Describe how quantitative variables were handled in the analyses (ie, scale, units, model).	**Text S2**
	b	Describe how genetic variants were handled in the analyses and, if applicable, how their weights were selected.	**N/A**
	c	Describe the MR estimator (eg, 2-stage least squares, Wald ratio) and related statistics. Detail the included covariates and, in case of 2-sample MR, whether the same covariate set was used for adjustment in the 2 samples.	**Page 6, 7**
	d	Explain how missing data were addressed.	**N/A**
	e	If applicable, indicate how multiple testing was addressed.	**N/A**
7	Assessment of assumptions	Describe any methods or prior knowledge used to assess the assumptions or justify their validity.	**Page 6, 7,**
8	Sensitivity analyses and additional analyses	Describe any sensitivity analyses or additional analyses performed (eg, comparison of effect estimates from different approaches, independent replication, bias analytic techniques, validation of instruments, simulations).	**Page 6-8**
9	Software and preregistration		
	a	Name statistical software and package(s), including version and settings used.	**Page 8**
	b	State whether the study protocol and details were preregistered (as well as when and where).	**N/A**
**Results**			
10	Descriptive data		
	a	Report the numbers of individuals at each stage of included studies and reasons for exclusion. Consider use of a flow diagram.	**Page 5**<br>**Table S1, S2**
	b	Report summary statistics for phenotypic exposure(s), outcome(s), and other relevant variables (eg, means, SDs, proportions).	**Table S3, S4**
	c	If the data sources include meta-analyses of previous studies, provide the assessments of heterogeneity acrossthese studies.	**N/A**
	d	For 2-sample MR:<br>i. Provide justification of the similarity of the genetic variant–exposure associations between the exposure and outcome samples.<br>ii. Provide information on the number of individuals who overlap between the exposure and outcome studies.	**N/A**
11	Main results		
	a	Report the associations between genetic variant and exposure and between genetic variant and outcome, preferably on an interpretable scale.	**Page 8**<br>**Table S5-S25**
	b	Report MR estimates of the relationship between exposure and outcome and the measures of uncertainty from the MR analysis, on an interpretable scale, such as odds ratio or relative risk per SD difference.	**Page 8-10**
	c	If relevant, consider translating estimates of relative risk into absolute risk for a meaningful time period.	**N/A**

	d	Consider plots to visualize results (eg, forest plot, scatterplot of associations between genetic variants and outcome vs between genetic variants and exposure).	**Page 9, 10**<br>**Figure 2-4**<br>**Figure S2-S17**
12	Assessment of assumptions		
	a	Report the assessment of the validity of the assumptions.	**Page 8-10,**
	b	Report any additional statistics (eg, assessments of heterogeneity across genetic variants, such as I^2, Q statistic, or E-value).	**Page 9, 10**<br>**Table1**<br>**Table S26, S30**
13	Sensitivity analyses and additional analyses		
	a	Report any sensitivity analyses to assess the robustness of the main results to violations of the assumptions.	**Page 9, 10**<br>**Figure S1, S10**
	b	Report results from other sensitivity analyses or additional analyses.	**Page 9-11,**
	c	Report any assessment of the direction of the causal relationship (eg, bidirectional MR).	**Page 9, 10**<br>**Table 2**<br>**Table S27**
	d	When relevant, report and compare with estimates from non-MR analyses.	**N/A**
	e	Consider additional plots to visualize results (eg, leave-one-out analyses).	**Page 10**<br>**Figure 5, 7**<br>**Figure S15-19**
**Discussion**			
14	Key results	Summarize key results with reference to study objectives.	**Page 11, 12**
15	Limitations	Discuss limitations of the study, taking into account the validity of the IV assumptions, other sources of potential bias, and imprecision. Discuss both direction and magnitude of any potential bias and any efforts to address them.	**Page 14, 15**
16	Interpretation		
	a	Meaning: Give a cautious overall interpretation of results in the context of their limitations and in comparison with other studies.	**Page 12, 13**
	b	Mechanism: Discuss underlying biological mechanisms that could drive a potential causal relationship between the investigated exposure and the outcome, and whether the gene-environment equivalence assumption is reasonable. Use causal language carefully, clarifying that IV estimates may provide causal effects only under certain assumptions.	**Page 13**
	c	Clinical relevance: Discuss whether the results have clinical or public policy relevance, and to what extent they inform effect sizes of possible interventions.	**Page 13, 14**
17	Generalizability	Discuss the generalizability of the study results (a) to other populations, (b) across other exposure periods/timings, and (c) across other levels of exposure.	**Page 15**
**Other Information**			
18	Funding	Describe sources of funding and the role of funders in the present study and, if applicable, sources of funding for the databases and original study or studies on which the present study is based.	**Page 23**
19	Data and data sharing	Provide the data used to perform all analyses or report where and how the data can be accessed, and reference these sources in the article. Provide the statistical code needed to reproduce the results in the article or report whether the code is publicly accessible and, if so, where.	**Page 22, 23**
20	Conflicts of interest	All authors should declare all potential conflicts of interest.	**Page 23**

Supplementary Methods S1. Detailed information about the database involved in this study

UK Biobank is a highly valuable biomedical database that provides detailed genetic and health information of 500,000 participants primarily aged 40 to 69 from the United Kingdom. It facilitates research on the most common and life-threatening diseases for researchers worldwide. UK Biobank collected blood, urine, and saliva samples from participants, as well as detailed lifestyle and health record information, during the period from 2006 to 2010. These data are anonymized and regularly augmented with additional data, such as genomic analysis, accelerometer, and multimodal imaging data. The majority of participants for most phenotypes in this study are derived from the UK Biobank.

Finngen is a nationwide GWAS research project in Finland that began collecting and analyzing genomic and health data from nearly 600,000 Finnish individuals since 2017. It is matched with digital health records generated by the National Health Registry. To this day, the number of phenotypes and sample size in Finngen continues to increase on a regular basis. FinnGen has leveraged the unique advantage of the Finnish population and has already discovered numerous novel genes that contribute to improving human health. The summary-level data for hypertension, heart failure, atrial fibrillation, and atrial flutter in this study are derived from the latest release of Finngen's R9 data in May 2023, which ensures avoidance of sample overlap with exposure GWAS data.

The IEU OpenGWAS project is a database developed by the Medical Research Council Integrative Epidemiology Unit (IEU-MRC) at the University of Bristol in the United Kingdom. It collects and analyzes summary-data from various global genome-wide association studies (GWAS) for querying or downloading purposes. The purpose of the IEU OpenGWAS project is to provide a high-quality, efficient, and reproducible source of GWAS data for researchers worldwide, enabling causal inference, fine mapping, colocalization, and other analytical methods related to human phenotypes and diseases.

Supplementary Methods S2. Describing how to handle GWAS summary-level data of variables

The GWAS summary-level data for all continuous phenotype report the effect sizes in one-standard-deviation (1-SD) change. The values of VLDL-C have undergone natural-log-transformation, while the values of TC have undergone inverse-normal-transformation. The effect size and their standard error for GWAS summary-level data of high cholesterol and hypercholesterolemia were computed using BOLT-LMM linear regression. Therefore, we transformed them into log odds using the following formula: $\log odds = \frac{\beta_{bolt}}{\mu(1-\mu)}$, and $se(\log odds) = \frac{se_{bolt}}{\mu(1-\mu)}$, where $\mu = n_{case}/(n_{case} + n_{control})$ is the case prevalence. The statistical analysis of GWAS summary-level data for other dichotomous phenotypes was conducted using logit regression model, and the effect sizes were computed in units of log odds.

Supplementary Methods S3. MRlap introduction and method description

MRlap accounts and corrects for biases arising from sample overlap, weak instrumental variables, and the winner's curse, using cross-trait LD-score regression (LDSC) to approximate sample overlap. If the corrected effect is not significantly different from the observed effect, then the effect estimate from the IVW method is reliable. Since MRlap requires correcting for the aforementioned biases, it necessitates the retention of a larger number of instrumental variables to enhance the precision and stability of estimates. However, when using default parameters, due to the relatively limited number of SNPs and the absence of partial SNP ID information in the outcome GWAS summary-level data, many SNPs are missing in the outcome summary-level data, resulting in unreliable results. So, we adjusted the parameters of MRlap, namely MR_threshold = 5e-08, MR_pruning_dist = 500, MR_pruning_LD = 0.001, MR_reverse = 1e-3, to retain as many SNPs as possible without losing any.
